# A global analysis of genetic interactions in *Caenorhabditis elegans*

**DOI:** 10.1186/jbiol58

**Published:** 2007-09-26

**Authors:** Alexandra B Byrne, Matthew T Weirauch, Victoria Wong, Martina Koeva, Scott J Dixon, Joshua M Stuart, Peter J Roy

**Affiliations:** 1 Department of Medical Genetics and Microbiology, The Terrence Donnelly Centre for Cellular and Biomolecular Research, 160 College St, University of Toronto, Toronto, ON, M5S 3E1, Canada; 2 Collaborative Program in Developmental Biology, University of Toronto, Toronto, ON, M5S 3E1, Canada; 3 Department of Biomolecular Engineering, 1156 High Street, Mail Stop SOE2, University of California, Santa Cruz, CA 95064, USA

## Abstract

**Background:**

Understanding gene function and genetic relationships is fundamental to our efforts to better understand biological systems. Previous studies systematically describing genetic interactions on a global scale have either focused on core biological processes in protozoans or surveyed catastrophic interactions in metazoans. Here, we describe a reliable high-throughput approach capable of revealing both weak and strong genetic interactions in the nematode *Caenorhabditis elegans*.

**Results:**

We investigated interactions between 11 'query' mutants in conserved signal transduction pathways and hundreds of 'target' genes compromised by RNA interference (RNAi). Mutant-RNAi combinations that grew more slowly than controls were identified, and genetic interactions inferred through an unbiased global analysis of the interaction matrix. A network of 1,246 interactions was uncovered, establishing the largest metazoan genetic-interaction network to date. We refer to this approach as systematic genetic interaction analysis (SGI). To investigate how genetic interactions connect genes on a global scale, we superimposed the SGI network on existing networks of physical, genetic, phenotypic and coexpression interactions. We identified 56 putative functional modules within the superimposed network, one of which regulates fat accumulation and is coordinated by interactions with *bar-1*(*ga80*), which encodes a homolog of β-catenin. We also discovered that SGI interactions link distinct subnetworks on a global scale. Finally, we showed that the properties of genetic networks are conserved between *C. elegans *and *Saccharomyces cerevisiae*, but that the connectivity of interactions within the current networks is not.

**Conclusions:**

Synthetic genetic interactions may reveal redundancy among functional modules on a global scale, which is a previously unappreciated level of organization within metazoan systems. Although the buffering between functional modules may differ between species, studying these differences may provide insight into the evolution of divergent form and function.

## Background

A basic premise of genetics is that the biological role of a gene can be inferred from the consequence of its disruption. For many genes, however, genetic disruption yields no detectable phenotype in a laboratory setting. For example, approximately 66% of genes deleted in *Saccharomyces cerevisiae *have no obvious phenotype [1]. A similar fraction of genes in *Caenorhabditis elegans *is also expected to be phenotypically wild type [2-4]. Elucidating the function of these genes therefore requires an alternative approach to single gene disruption.

One way to uncover biological roles for phenotypically silent genes is through genetic modifier screens. Genetic modifiers are traditionally identified through a random mutagenesis of individuals harboring one mutant gene followed by a screen for second-site mutations that either enhance or suppress the primary phenotype (reviewed in [5]). Modifying genes identified in this way clearly participate in the regulation of the process of interest, yet often have no detectable phenotype on their own [6-10]. Thus, forward genetic modifier screens are a useful but indirect approach to ascribe function to genes that otherwise have no phenotype.

An elegant approach called synthetic genetic array (SGA) analysis was devised to systematically analyze the phenotypic consequences of double mutant combinations in *S. cerevisiae *[11]. With SGA, a 'query' deletion strain is mated to a comprehensive library of the nonessential deletion strains [1] through a mechanical pinning process. Resulting double-mutant combinations typically have growth rates indistinguishable from single-mutant controls. However, some deletion pairs produce a 'synthetic' sick or lethal phenotype not shared by either single mutant, indicating a genetic interaction. The revelation that most nonessential genes synthetically interact with several partners from different pathways [11,12] was a major biological insight, as it suggests that many genes have multiple redundant functions and provides a satisfying explanation for the apparent lack of phenotype for the majority of gene disruptions. Other SGA-related techniques have been devised to investigate interactions with essential genes [13] and to mine the consequences of interactions in great detail [14]. An alternative approach to SGA has been developed to create double mutants *en masse *by transforming the entire deletion library in liquid with a transgene that targets a query gene for deletion [15].

Synthetic interactions can reveal several classes of genetic relationships. First, disrupting a pair of genes that belong to parallel pathways that regulate the same essential process may reveal a 'between-pathway' interaction. Second, compromising a pair of genes that act either at the same level of the pathway or are ancillary components at different levels of the pathway may reveal a 'within-pathway' interaction. Finally, each gene of an interacting pair may act in unrelated processes that collapse the system when compromised together through poorly understood mechanisms, revealing an 'indirect' interaction [16]. We note that as the cell may function by coordinating collections of gene products that work together as discrete units, called molecular machines or functional modules [17,18], these 'indirect interactions' may actually reveal redundancy between previously unrecognized functional modules. To investigate which model best describes an interaction in yeast, physical-interaction data have been mapped onto synthetic genetic-interaction networks [11,12,16,19]. This type of analysis suggests that between-pathway models account for roughly three and a half times as many synthetic genetic interactions compared with 'within-pathway' models.

Although the tools that accompany *S. cerevisiae *as a model system make it ideal for genome-wide analyses of genetic interactions in a single-celled organism, we wanted to apply a similar systematic approach towards a global understanding of genetic interactions in an animal. There is, however, no comprehensive collection of mutants, null or otherwise, in any animal model system. Notwithstanding this, several features make the nematode worm *Caenorhabditis elegans *uniquely suited among animal model systems to systematically investigate genetic interactions in a high-throughput manner. First, the worm has only a three-day life cycle. Second, animals can be easily cultured in multiwell-plate format, making the preparation of large numbers of samples economical. Third, around 99.8% of the individuals within a population are hermaphrodites. Strains therefore propagate during an experiment without the need for human intervention. Fourth, genes can be specifically targeted for reduction-of-function through RNA interference (RNAi) by feeding [20]. A library of *Escherichia coli *strains has been generated in which each strain expresses double-stranded (ds) RNA whose sequence corresponds to a particular worm gene. Upon ingesting the *E. coli*, the dsRNAs are systemically distributed and target a particular gene for a reduction-of-function by RNAi [21]. RNAi-inducing bacterial strains targeting over 80% of the 20,604 protein-coding genes of *C. elegans *have been generated [3,22]. Another useful feature of the worm is the large collection of publicly available mutants representing most of the conserved pathways that control development in all animals [23]. Together, these features make *C. elegans *a unique whole-animal model to systematically probe genetic interactions in a high-throughput fashion.

Here, we describe a novel approach towards a global analysis of genetic interactions in *C. elegans*. Our approach is called systematic genetic interaction analysis (SGI) and relies on targeting one gene by RNAi in a strain that carries a mutation in a second gene of interest. The SGI approach is similar in principle to that used by Fraser and colleagues (Lehner *et al*. [24]), but with four key differences. First, Lehner *et al*. investigated interactions in liquid culture, whereas we carried out all experiments on the solid agar substrate commonly used by *C. elegans *geneticists. Second, rather than score population growth in a binary manner, we used a graded scoring scheme to measure population growth. Third, rather than test all potential interactions in side-by-side duplicates [24], we performed all experiments in at least three independent replicates in a blind fashion. Finally, we used a global analysis of our data to identify interacting gene pairs in an unbiased fashion. Using SGI analysis, we identified 1,246 interactions between 461 genes, which is the largest genetic-interaction network reported to date.

We present several lines of evidence showing that the SGI network meets or exceeds the quality of other large-scale interaction datasets. Analysis of the SGI network reveals new functions for both uncharacterized and previously characterized genes, as well as new links between well-studied signal transduction pathways. We integrated the SGI network with other networks and found that synthetic genetic interactions typically bridge different subnetworks, revealing redundancy between functional modules [18]. Finally, we provide evidence that the properties of the *C. elegans *synthetic genetic network are conserved with *S. cerevisiae*, but the network connectivity of the interactions differs between the two systems. Thus, SGI analysis not only reveals novel gene function, but also contributes to our understanding of genetic-interaction networks in an animal model system.

## Results

### Constructing the SGI network

To better understand how genes regulate animal biology on a global scale, we systematically tested genetic interactions between 11 'query' genes (Table 1) and 858 'target' genes (see Additional data file 1). Ten of the query genes belong to one of six signaling pathways specific to metazoans, including the insulin, epidermal growth factor (EGF), fibroblast growth factor (FGF), Wingless (Wnt), Notch, and transforming growth factor beta (TGF-β) pathways (see Table 1). The 11th query gene, *clk-2*, is a member of the DNA-damage response (DDR) pathway and is included in our analysis as an example of a gene not involved in the transduction of a signal from the plasma membrane. The 858 target genes consist of 372 genes that are probably involved in signal transduction from the plasma membrane on the basis of their annotation in Proteome (BIOBASE, Wolfenbüttel, Germany) [25], and 486 genes from linkage group III from which new signaling genes might be identified. We will henceforth refer to these groups of genes as the 'signaling targets' and the 'LGIII targets', respectively. An analysis of the LGIII set suggests that the 486 genes are random with respect to known functional categories (*p *> 0.05) (see Materials and methods and Additional data file 2). All of the queries were tested against the signaling targets, and six of the queries, representing five pathways, were tested against the LGIII targets (see Table 1).

**Table 1:** A summary of the query genes

Query gene	Ortholog (pathway)	Null/strong loss-of-function phenotype(s)	Hypomorphic phenotype(s)	References
*let-756*	FGF (FGF)	Early larval arrest (*s2887*)	scrawny, Slo (*s2613*)**	[77]
*egl-15*	FGF receptor (FGF)	Early larval arrest (*n1456*)	scrawny, Egl (*n1477*)**	[78]
*let-23*	EGF receptor (EGF)	L1 arrest (*mn23*)	ts Vul, pleotropic (*n1045*)**	[79]
*daf-2*	Insulin growth factor receptor (insulin)	Emb (*e979*)	ts Daf-c (*e1370*)**	[35]
*sem-5*	GRB-2 (EGF, FGF, insulin)	L1 arrest (leaky) (*n1619*)	Egl, Vul (*n2019*)*	[79,80]
*sos-1*	Guanine-nucleotide exchange factor (EGF, FGF)	Emb (*s1031*)	ts Egl, Vul (*cs41*)*	[33]
*let-60*	RAS (EGF, FGF, insulin, Wingless/Wnt)	Mid-larval lethal (leaky) (*s1124*)	Egl, Vul (*n2021*)*	[81,82]
*glp-1*	Notch receptor (Notch)	ts Emb (*gp60*)	ts Emb, Glp, Muv (*or178*)*	[47]
*bar-1*	β-catenin (Wingless (Wnt))	Mig, Vul, Pvl (*ga80*)**	Mig, Vul, Pvl (*mu63*)	[34]
*sma-6*	Type I TGF-β receptor (TGF-β)	Sma, Mab (*wk7*)	Sma (*e1482*)*	[83]
*clk-2*	Tel-2p (DNA-damage response)	Unknown	Slo, Ste, ts Emb (*mn159*)**	[84]

In the second column, 'ortholog' refers to the canonical ortholog in yeast, flies, mice, or humans. The pathway to which the ortholog belongs is in brackets. Third column: if known, the null or strong loss-of-function phenotype is shown. Fourth column: weak loss-of-function (hypomorphic) phenotypes are shown for representative alleles. Phenotypic acronyms: Emb, embryonic lethal; Daf-c, dauer formation constitutive; Slo, slow growth; Egl, egg-laying defective; Vul, vulvaless; Glp, germ-line proliferation defects; Muv, multivulva; Mig, cell and/or axon migration defects; Pvl, protruding vulva; Sma, small body; Mab, male tail abnormal; Ste, sterile; ts, temperature sensitive. The alleles used in this study are followed by two asterisks if used as a query against both the signaling targets and the LGIII targets, or just a single asterisk if used only against the signaling targets.

**Figure 1:**
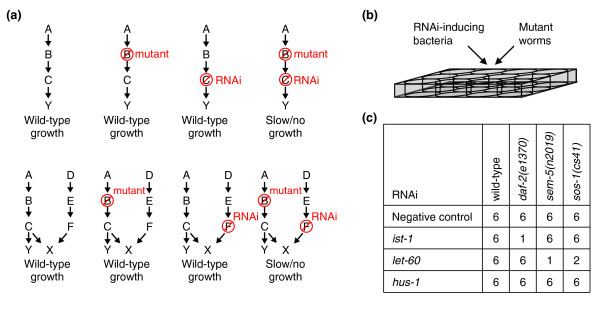
Synthetic genetic-interaction (SGI) analysis in *C. elegans*. **(a) **Two scenarios that may result in synthetic interactions are presented. The top row shows how enhancing interactions may arise when hypomorphic loss-of-function worms (mutant), which have reduced but not eliminated function of a gene, are fed RNAi that targets another gene in the same essential pathway. The lower row shows synthetic interactions that may arise when a hypomorph and a gene targeted by RNAi are in parallel pathways that regulate an essential process (X). **(b) **An outline of the SGI experimental approach. RNAi-inducing bacteria that target a specific *C. elegans *gene for knockdown (target gene A) are fed to a hypomorphic mutant (query gene B). In parallel, wild-type worms are fed the experimental RNAi-inducing bacteria (control 1), and the query mutant is fed mock RNAi-inducing bacteria (control 2). This is all done in 12-well plate format with at least three technical replicates. Over the course of several days, we estimate the number of progeny produced in each experimental and control well in a blind fashion (see text and Materials and methods). We assigned a growth score from 0–6 (0, 2 parental worms; 1, 1–10 progeny; 2, 11–50 progeny; 3, 51–100 progeny; 4, 101–200 progeny; 5, 200+ progeny; and 6, overgrown). **(c) **Interacting gene pairs are inferred through a difference in the population growth scores between experimental and control wells. In the example shown, a global analysis of the experimental and control query-target combinations revealed that *daf-2 *interacts with *ist-1*, and that *sem-5 *and *sos-1 *both interact with *let-60*.

To systematically test for genetic interactions between query-target pairs, worms harboring a weak loss-of-function mutation in a query gene were targeted for RNAi-mediated reduction of function in a second (target) gene by feeding the appropriate dsRNA [3,20,21]. We estimated the number of progeny resulting from each query-target combination and compared the counts to controls (Figure 1, and see Materials and methods). We expected that if the query and target interacted, the resulting number of progeny would be lower than wild-type (N2) worms fed the target RNAi (control 1) or the query mutant worms fed mock-RNAi (control 2). Each query-target pair was tested at least in triplicate on solid agar substrate in 12-well plates. We estimated the number of resulting progeny in each well over the course of several days as the progeny matured, and assigned each well a score from zero to six. For example, wells containing no progeny received a score of zero, whereas wells overgrown with progeny were given a score of six.

**Figure 2:**
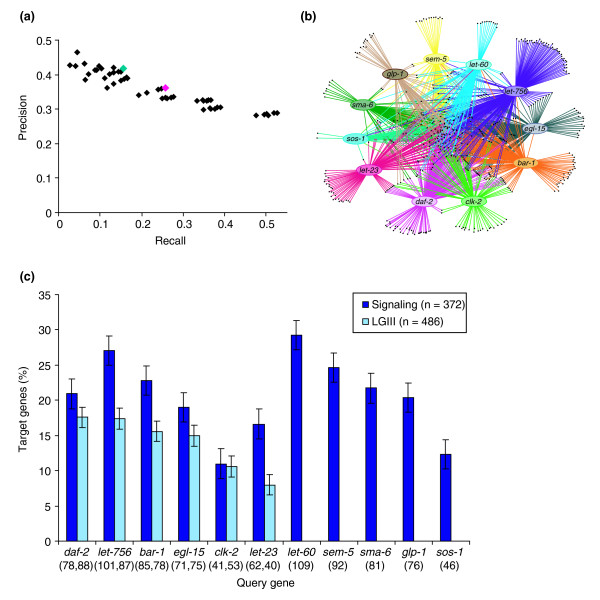
The SGI network. **(a) **The precision and recall of the 51 unique network variants, as calculated with respect to GO Biological Process annotation (see Materials and methods). The high-confidence variant is highlighted in pink and the SGI variant in teal. **(b) **The SGI network contains 1,246 unique synthetic genetic interactions, of which 833 (67%) are between a query gene and a gene in the signaling set, and 413 (33%) are between a query gene and a gene in the LGIII set. Visualization generated with Cytoscape [85]. **(c) **The percentage of target interactions per query gene in both the signaling (dark-blue) and the LGIII (light-blue) networks. The raw number of interacting target genes in each experiment (signaling, LGIII) is shown below each bar. The error bars represent one standard deviation assuming a binomial distribution.

We developed an unsupervised computational method based on reproducibility and the nature of the population scores in order to determine objectively which query-target pairs interact genetically. We first arrayed the target genes plus control 1 on one axis, and the query genes plus control 2 on the other axis to create a matrix of 56,347 scores that included all experimental replicates over several days. We then identified six different attributes that could be mined to infer a unique set of genetic interactions from the matrix. Some of these attributes include the reproducibility of scores among technical replicates, the consistency of scores over each day of observation, and the difference in the scores between the experimental gene pair and controls (see Materials and methods). By varying the selection parameters for each attribute, we identified 51 unique variant sets of interactions or networks (Figure 2a).

To identify the network variant that maximized the number of likely true positives but minimized the number of likely false positives, we first identified those interacting pairs that share the same Gene Ontology (GO) biological process [26] (see Materials and methods). We calculated 'recall' for each variant by dividing the number of co-classified interacting pairs by the number of all possible co-classified pairs within the variant. Similarly, we calculated 'precision' by dividing the number of co-classified interacting pairs by the total number of interacting pairs in the variant. A variant with high recall and low precision is likely to have good recovery of all possible co-classified genetic interactions, but its low stringency will result in a high number of false positives. On the other hand, a network with low recall and high precision will have a low number of false positives, but may have a greater number of false negatives. As is evident from the recall and precision plot (see Figure 2a), there are several network variants with high recall and precision values. We estimated the significance of the extent to which each variant network links genes in the same GO biological process using the hypergeometric distribution (see Materials and methods). Henceforth, we denote *p *values calculated using the hypergeometric distribution with 'hg'. The most significant variant contains 656 unique interactions among 253 genes (*p *< 10^-22^)^hg ^and has a precision and recall of 42% and 16%, respectively. The next best variant (*p *< 10^-21^)^hg ^contains nearly twice as many interactions (1,246) among 461 genes, and has 10% higher recall. We chose to restrict all further analysis to the latter network in order to capture more previously uncharacterized interactions. We refer to this variant as the SGI network (Figure 2b, and Additional data file 3). All 656 interactions within the smaller variant are contained within the SGI network and are hereafter referred to as 'high confidence SGI interactions'. The SGI network contains 833 interactions between query genes and signaling targets (67%), and another 421 between query genes and LGIII targets (33%). These 1,246 interactions range in strength from weak to very strong (Additional data file 4). Each of the 1,246 gene pairs within the SGI network synthetically interact by a conservative estimate, as the double gene perturbation phenotype is greater than the product of the two single gene perturbations (see Additional data file 5) [14,27]. All of the interactions fell within one interconnected component because each query gene shared interaction targets with at least one other query gene.

We assessed the reproducibility of SGI interactions by analyzing reciprocal and technical replicates. Reciprocal reproducibility was measured by interchanging the method used to downregulate each member of selected query-target gene pairs. Interacting query-target pairs were retested by targeting the query gene by RNAi in the background of a mutated 'target' gene. Six of the queries in our matrix were also included as RNAi targets, providing 15 gene pairs to test for reciprocity. All of the 15 gene pairs interacted in one test, and six (40%) also interacted in the reciprocal test (Additional data file 6). Reciprocity of 100% is not expected because mutations and RNAi experiments often differ in their effects on gene function [3,22,28]. We also measured the technical reproducibility of the assay. For technical replicates, 15 of the target genes and six of the query genes were included in both the signaling and LGIII matrices, providing replicates for 90 query-target pairs. Of these, eight are positive and 67 are negative in both sets, yielding a technical reproducibility of 83% (75/90). Together, these results demonstrate that SGI interactions are reproducible.

### A functional analysis of SGI interactions

All of the query genes included in this study, except *clk-2*, are required in signal transduction from the plasma membrane. *clk-2 *was included as a query gene in our screen to gauge the specificity of SGI interactions on a global scale. We expected that *clk-2 *would interact with fewer 'signaling' targets than would the signaling queries. In addition, we expected that *clk-2 *would interact with a similar number of signaling targets compared to LGIII targets, whereas the signaling queries would preferentially interact with other signaling genes. Indeed, we found that *clk-2 *interacts with half as many signaling genes compared with the average signaling query (11.0% versus 21.5%, respectively) and interacts with the fewest signaling targets overall (Figure 2c). By contrast, *let-60*, which encodes the *C. elegans *ortholog of the small GTPase Ras, interacts with the greatest number of signaling targets (29.2%), probably because of the pleiotropic function of Ras in signal transduction [29]. The fraction of LGIII targets that interact with signaling queries is 32% less than the fraction of signaling targets that interact with signaling queries (14.7% versus 21.5%). By contrast, the fraction of *clk-2 *interactions with signaling or LGIII targets is nearly identical (11.0% versus 10.6%, respectively). These results further support the validity of the SGI approach.

**Figure 3:**
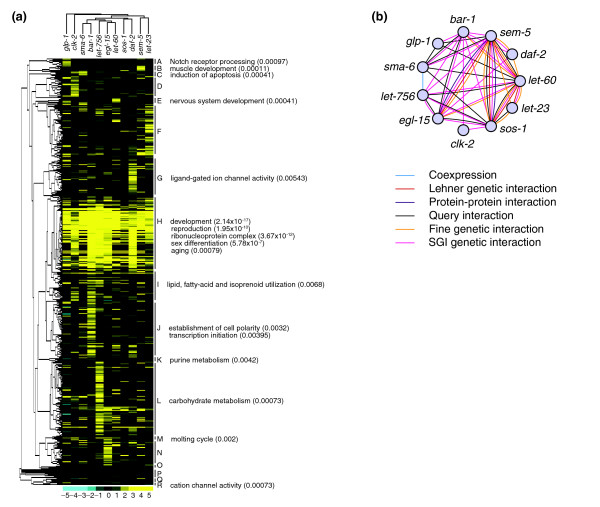
Global patterns of interactions within the SGI network. **(a) **Two-dimensional clustergram of SGI interactions based on average strength of interaction. RNAi-targeted genes are represented along the rows and the 11 query hypomorphs across the columns. The shades from black to yellow on the bottom scale indicate increasing interaction strength, and shades from black to light-blue indicate increasing alleviating interaction strength. Alleviating interaction strengths indicate that the double reduction-of-function worms grow better than controls. **(b) **The query network. Query genes (nodes) are linked in this network if they share a significant number of interaction partners or if there is evidence of a functional interaction (see text). Edges are colored according to the type of supporting evidence (see text and Materials and methods for more details). Visualization generated with Cytoscape [85].

Next, we exploited the graded scoring scheme used to collect SGI data to investigate patterns of interactions within the matrix of genetic-interaction tests. The strength of interaction between each tested gene pair was calculated based on the average difference between the experimental growth scores and the controls. The strength of interaction for each gene pair was then clustered in two dimensions to group queries and targets on the basis of similar growth patterns (see Materials and methods). Clusters of target genes were then examined for enrichment of shared functional annotation (Additional data file 7 and see Materials and methods). The resulting clustergram reflects the characterized roles of many genes and provides evidence supporting previously uncovered relationships (Figure 3a). For example, the first cluster of target genes is enriched for the annotation 'Notch receptor-processing', and is clustered on the basis of the phenotype of shared slow growth in a *glp-1 *mutant background, which has a mutant Notch receptor. Similarly, a cluster of genes enriched for 'establishment of cell polarity' predominantly interact with *bar-1 *(encoding a β-catenin homolog) (cluster J, Figure 3a). Also, a cluster of genes characterized by the phenotype of slow growth in a *clk-2*(*mn159*) background are enriched for 'induction of apoptosis' (cluster C, Figure 3a). Interestingly, genes in this group also have a slow-growth phenotype in a *sma-6 *(type I TGF-β receptor homolog) background. Although well characterized in other systems [30], this is the first reported evidence for a functional link between the TGF-β pathway and apoptosis in *C. elegans*. Finally, clusters of target genes with low growth scores in the background of many of the query mutants have general annotations such as 'reproduction' and 'aging'. This may reflect the involvement of many signaling pathways in these processes. Within all of these clusters are previously uncharacterized genes, which form the basis for numerous hypotheses.

To explore the connectivity between the EGF, FGF, Notch, insulin, Wnt, and TGF-β signaling pathways, we analyzed the SGI data in three ways. First, we examined the clusters of query genes on the clustergram and found some expected patterns, including the grouping of the genes for the FGF receptor (*egl-15*), its ligand (*let-756*), and their downstream mediator (*let-60*/RAS) (Figure 3a). As expected, *clk-2 *and *glp-1 *do not cluster with the receptor tyrosine kinases or their downstream mediators. By contrast, *sma-6 *and *bar-1*/β-catenin are closely linked, suggesting cooperation between TGF-β and the Wnt/β-catenin pathways, as previously reported in other organisms [31]. Second, we investigated the connectivity between the signaling pathways by creating a network of query genes (Figure 3b, and Additional data file 3). Because six of the query mutants were also included as RNAi targets within the SGI matrix, we tested query pairs directly for interactions and found 25 interactions among 45 pairs. In addition, we examined the pattern of interactions between each query gene and the entire set of RNAi targets. Functionally related query genes are expected to interact with an overlapping set of target genes [11,12,32]. We therefore connected queries within the query network with a 'congruent' link if they shared interactions with the same targets more frequently than expected by chance (*p *< 10^-9^)^hg ^(see Materials and methods). As expected, the proximity of query genes to each other in the clustergram is reflected in the congruent links. Finally, we added links to the query network derived from other datasets considered throughout this study. These included protein-protein interactions, coexpression links, phenotype links, and other genetic data, all of which are described in detail below. The resulting query network contains 11 nodes and 33 query-query interactions, 16 of which are supported by multiple sources. Of the 24 SGI links within the query network, eight are supported by other lines of evidence that include previously described genetic interactions between genes within defined pathways. Therefore, 16 of the SGI links represent previously unreported interactions, seven of which are also supported by congruent links.

Many of the interaction patterns within the query network are expected. For example, the downstream mediators of receptor tyrosine kinase signaling (*let-60*, *sem-5 *(homologous to the human gene encoding the adaptor protein GRB2), and *sos-1 *(encoding a homolog of the SOS2 adaptor protein)) have the highest number of links within the query network (21, 21, and 18 respectively). This pattern is expected given that almost half of the pathways analyzed involve receptor tyrosine kinase signaling. Interestingly, *let-60 *and *sem-5 *each interact with all of the query genes but do not interact with *clk-2*, suggesting that they are common mediators of signal transduction. As expected, *clk-2 *has the fewest links. We also identified many multiply supported links between *let-23*, *let-60*, *sem-5*, and *sos-1*, which are previously characterized components of the EGF pathway [29,33]. Furthermore, previously characterized cross-talk between *let-60 *and *bar-1 *[34], and between *daf-2 *(encoding the insulin receptor) and *sem-5 *[35] is supported. The query network provides the first evidence of genetic interactions between the FGF gene *let-756 *and downstream mediators of the FGF pathway, including the FGF receptor gene *egl-15*, *let-60*, *sem-5*, and *sos-1*, affirming several previous lines of evidence [36]. Furthermore, *let-756 *and *egl-15 *each interact with six query genes, five of which are shared between the two. Finally, the query network reveals novel interactions between *bar-1 *and *glp-1*, between *bar-1 *and *sma-6*, and between *bar-1 *and multiple components of the FGF and EGF pathways. Further investigation will be required to elucidate the precise role of these interactions during development.

### A comparison of the SGI network with other networks

**Figure 4:**
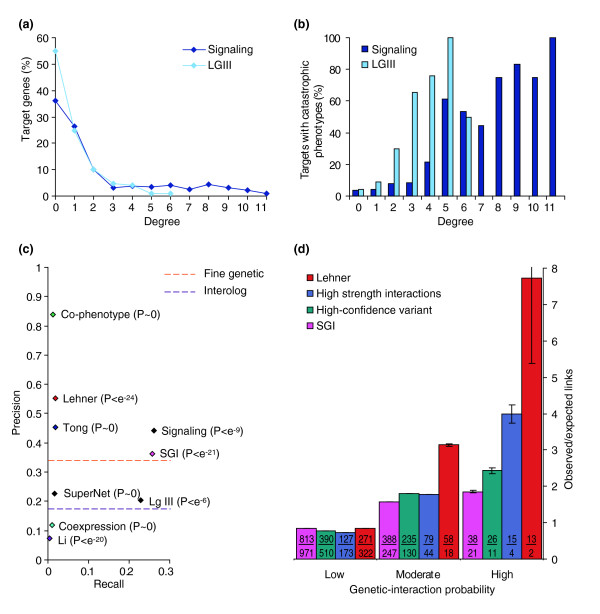
Network properties of SGI and other published datasets. **(a) **A plot of the percentage of targets (*y*-axis) that interact with a given number of query genes (*x*-axis), illustrating that the SGI network has properties similar to that of scale-free networks. **(b) **A plot of the percentage of targets that yield a catastrophic phenotype when targeted by RNAi in a wild-type background [3] (*y*-axis) as a function of how many query genes they interact with (degree, *x*-axis). **(c) **The precision and recall of interaction networks calculated with respect to GoProcess1000 (see Materials and methods). Significance values (in brackets) were calculated using the hypergeometric distribution. The source of the networks is presented in the text, except for the SuperNet (superimposed network, see Materials and methods). The orange dashed line indicates the precision of the fine genetic interactions extracted from WormBase. The lower dashed line indicates the precision of the interolog network (see Materials and methods). The recall of these two datasets cannot be calculated, as the number of genes that were tested cannot be ascertained. **(d) **An independent test of the likelihood of true interactions among the Lehner [24] and SGI genetic-interaction datasets using the algorithm of Zhong and Sternberg [44], which predicts a confidence level for a genetic interaction between any given gene pair in *C. elegans*. The 656 interactions of the 'high-confidence' SGI variant, along with the 229 interactions of the highest interaction strength within the SGI network are also analyzed. Each experimentally derived interacting gene pair is binned according to the confidence level predicted by Zhong and Sternberg (*x*-axis): low-, moderate- and high-confidence predictions have interaction probabilities of 0–0.6, 0.6–0.9, and 0.9–1.0, respectively. The results are plotted as a ratio of the number of experimentally identified interacting gene pairs to the number of gene pairs expected to be in that bin by chance (*y*-axis). Expected counts were determined by assuming a uniform distribution across all bins for all tested gene pairs. Values within each bar show the number of observed gene pairs over the number expected by chance. The key indicates the data source. Error bars indicate one standard error of the mean.

The analysis of large-scale interaction datasets from *C. elegans *provided pioneering insights into the nature of metazoan networks and demonstrated that network principles are conserved between yeast and worms [37-40]. Using the 1,246 genetic interactions of the SGI network, we asked if genetic network properties are also conserved. First, we found that SGI interactions have properties similar to scale-free networks: most SGI target genes interact with few query genes and few target genes interact with many query genes (Figure 4a). Second, we found that highly connected target genes, called hubs, within the SGI network are more likely to result in catastrophic phenotype when knocked-down by RNAi in a wild-type background compared with less connected targets (*p *< 10^-47^) (Figure 4b, and see Materials and methods). Third, we found that the average shortest path length (2.7 ± 0.8), clustering coefficient (0.3 ± 0.3), and average degree (5.4 ± 18.6) of the *C. elegans *genetic network are indistinguishable from those of the SGA synthetic genetic network, which has an average shortest path length of 3.3 ± 0.8, a clustering coefficient of 0.1 ± 0.2, and an average degree of 7.8 ± 16.9 [11,12] (see Materials and methods). These results demonstrate that the network properties of SGI are conserved with those of the yeast SGA network.

We next examined how the recall and precision of the SGI network compared with other large eukaryotic interaction networks, including a previously described *C. elegans *genetic-interaction network (Lehner *et al*. [24]), a *C. elegans *protein-interaction network (Li *et al*. [37]), a eukaryotic protein-interaction network that augments the *C. elegans *protein-interaction network with orthologous interactions from *S. cerevisiae*, *Drosophila melanogaster*, and human protein interactions contained in BioGRID [41], an mRNA coexpression network constructed from *C. elegans*, *S. cerevisiae*, *D. melanogaster*, and human expression data [38,40], an *S. cerevisiae *synthetic genetic-interaction network (Tong *et al*. [12]), and a network we created based on the similarity of *C. elegans *RNAi-induced phenotypes [3,4,22,42] (Figure 4c, and Materials and methods). We refer to these networks as the Lehner, Li, interolog, coexpression, Tong, and co-phenotype networks, respectively. In addition, we examined a network of fine genetic interactions, which consists of genetic interactions identified from low-throughput experiments that were collected from the literature by WormBase [43]. The fine genetic network excludes interactions identified solely through high-throughput analysis. The SGI network has an average precision, but a higher recall than all other datasets examined. We investigated whether the SGI network has a higher recall because of a preselection of signaling target genes, but found this not to be true: the recall of the SGI network remains the highest of all networks examined when only the LGIII target genes are considered (recall = 0.23). Together, our analyses suggest that the SGI approach is at least as proficient as other efforts that describe interactions on a large scale.

Next, we compared the SGI interactions to those found in the Lehner genetic-interaction network (Table 2). Of the 6,963 gene pairs tested for interaction by SGI, 1,165 were also tested by Lehner *et al*. [24]. Of these, 78.5% do not interact in either study. Of the 28 pairs found to interact by Lehner *et al*., 18 also interact in the SGI network. There are no obvious differences in the phenotypes of the 18 interacting gene pairs found in both the Lehner and SGI sets, compared with the 10 pairs found only in the Lehner set [3]. Overall, SGI identifies 64.3% of Lehner interactions and there is 98.9% concordance of the negative calls (*p *< 10^-27^). Of the 1,165 pairs tested by both screens, the SGI approach identified 222 additional interactions. The gene pairs that only interact in SGI are as likely to connect genes with shared GO annotation as are gene pairs that only interact in the Lehner network, as measured by precisions of 0.66 and 0.60, respectively. These observations suggest that both approaches can identify genetic interactions with equal precision, but that SGI captures more interactions.

**Table 2:** Comparison of SGI and Lehner genetic interactions

Type of link	Number of links*
Tested in SGI and Lehner analyses	1,165
Negative in SGI and Lehner analyses	915 (78.5%)
Positive in SGI and Lehner analyses	18 (1.5%)
Positive only in SGI analysis	222 (19.1%)
Positive only in Lehner analysis	10 (0.85%)

*Percentage of gene pairs tested in both SGI and Lehner analyses.

We extended the comparison between the SGI and Lehner networks by using previously computed prediction scores for *C. elegans *genetic interactions based on characterized physical interactions, gene expression, phenotypes, and functional annotation from *C. elegans*, *D. melanogaster*, and *S. cerevisiae *(Zhong and Sternberg [44]). The probability scores assigned by Zhong and Sternberg for all pairs of genes in the SGI network were divided into three categories: low probability of interaction; intermediate probability of interaction; and high probability of interaction. We found roughly twice as many SGI interactions as expected in the high-probability category and fewer gene pairs than expected in the low probability of interaction category (*p *< 10^-25^) (Figure 4d). The 'high confidence' SGI interactions have more high probability scores than expected compared with the whole SGI network (see Figure 2a), and the SGI interactions with the greatest interaction strengths (greater than 4.4) have more still. The Lehner genetic interactions have the greatest number of high-probability interactions relative to that expected by chance. As Lehner *et al*. [24] exclusively scored catastrophic interactions, this analysis suggests that the Zhong and Sternberg probability score not only reflects the likelihood of interaction, but also the strength of that interaction. Together, our comparison of SGI interactions to other observed and predicted networks further supports confidence in SGI interactions.

### Genetic interactions are orthogonal to other interaction datasets

We next asked how worm genetic interactions relate to other interaction datasets and how this adds to our understanding of systems in animals. To do so, we first created a superimposed network by combining published interaction data from numerous sources using a method similar to that used in [45]. We then investigated the patterns of SGI interactions within it. The superimposed network was constructed from several large-scale interaction datasets, including the Li, interolog, Lehner, coexpression, co-phenotype, and fine genetic-interaction networks (see above). In addition, the SGA network [12] was mapped onto *C. elegans *orthologs and is referred to as the 'transposed SGA network' (see Materials and methods). The links from all of these networks were combined with the SGI network to form a single superimposed network.

Altogether, the superimposed network contains 7,825 genes connected by 75,283 links: 43,363 eukaryotic coexpression links, 2,620 previously reported *C. elegans *genetic interactions, 7,527 transposed synthetic genetic interactions from yeast, 12,796 eukaryotic protein-protein interactions, 3,967 *C. elegans *protein-protein interactions, 8,862 co-phenotype links, and 1,246 SGI links (see Additional data file 3). Only 1.2% of the interactions within the superimposed network are supported by multiple data types (Table 3). Concomitantly, there is little overlap between any genetic-interaction dataset and other modes of interaction, suggesting that genetic interactions typically reveal novel relationships between genes.

**Table 3:** Composition of the *C. elegans *superimposed network

Network	Links	Nodes	Supported links	Genetically supported links (A)	Genetically supported links (B)	Physically supported links	Coexpression supported links	Co-phenotype supported links
Superimposed network	75,283	7,825	929 (7.2)	NA	NA	NA	NA	NA
SGI	1,246	461	63 (2.0)	43 (1.6)	53 (1.8)	9 (5.6)	2 (9.0)	4 (5.9)*
Lehner	341	161	25 (5.5)	13 (10.8)	23 (7.3)	3 (22.7)	1 (17.9)	1 (30.3)
Fine genetic interactions	2,279	1,022	152 (4.6)	NA	48 (1.7)	61 (27.8)	23 (36.1)	22 (20.2)
Transposed SGA	7,527	426	66 (2.3)	5 (4.5)	5 (3.2)*	43 (2.2)	14 (3.0)	4 (1.3)*
Interolog	12,796	4,339	723 (9.9)	61 (27.8)	110 (4.8)	NA	577 (14.6)	42 (3.9)
*C. elegans *protein interaction	3,967	2,624	27 (3.7)	7 (10.6)	10 (4.2)	NA	13 (3.8)	5 (3.4)*
Eukaryotic coexpression	43,363	5,232	695 (11.8)	23 (36.1)	40 (7.2)	577 (14.6)	NA	84 (6.1)
*C. elegans *co-phenotype	8,862	913	153 (5.2)	22 (20.2)	30 (6.1)	42 (3.9)	84 (6.1)	NA

The supported links column gives the number of links supported by other data within the superimposed network. The fold-enrichment over the average number obtained from 1,000 randomly permuted superimposed networks (representation factor) is given in brackets. Genetically supported links (A) refers to the number of links supported by fine genetic analysis reported in WormBase (release 170). Genetically supported links (B) refers to the number of links supported by genetic interactions reported in WormBase (release 170), Lehner *et al*. [24] or SGI. Physically supported links refers to the number of links supported by eukaryotic physical interactions (interologs; see text for details). Coexpression-supported links refers to the number of links supported by eukaryotic mRNA coexpression analysis (see text for details). Co-phenotype-supported links refers to the number of links supported by *C. elegans *co-phenotype correlations (see text for details). Unless followed by an asterisk, *P *values of the representation factor < 10^-4^. NA, not applicable.

**Figure 5:**
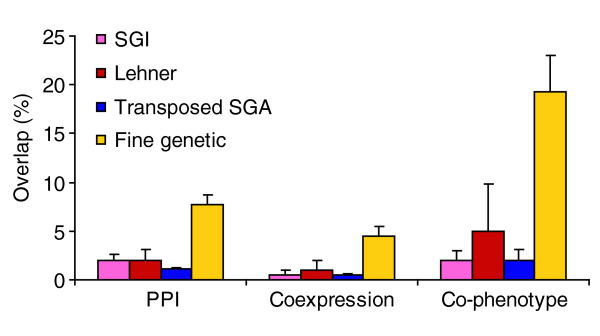
An analysis of the overlap between genetic interactions and other modes of interaction. The number of genetically interacting gene pairs from SGI, Lehner [24], the transposed SGA dataset [12] and low-throughput 'fine genetic interactions' [43] (see text and Materials and methods) that also interacted through direct protein-protein interactions (PPI) [37], or were tightly coexpressed (coexpression) [38,40], or had similar phenotypic profiles (co-phenotype) [3,4,42] (see Materials and methods) was analyzed (*x*-axis). Only gene pairs tested in both relevant datasets are considered here. To account for the differences and disparity of genes tested in the various screens, the results are represented as the number of interactions that overlap between the two datasets as a function of the number of identical or homologous gene pairs tested in both studies (*y*-axis). Error bars indicate one unit of standard deviation assuming a binomial distribution.

We next investigated the overlap between genetic interactions and other types of data within the superimposed network. We found that fine genetic interactions are supported by far more physical interactions when compared with SGA interactions (Figure 5), consistent with the idea that fine genetic interactions are enriched for 'within-pathway' interactions and that SGA interactions are enriched for 'between-pathway' interactions [12,16,19]. We found that the fraction of SGI and Lehner genetic interactions supported by physical interactions is indistinguishable from the fraction of SGA links supported by physical interactions (see Figure 5). Similar results were obtained when the analysis was repeated to measure the proportion of genetically interacting gene pairs that overlap with either the coexpression or co-phenotype networks (see Figure 5). We therefore conclude that the SGI and Lehner genetic interactions are probably biased towards between-pathway interactions, similar to those revealed by SGA.

**Figure 6:**
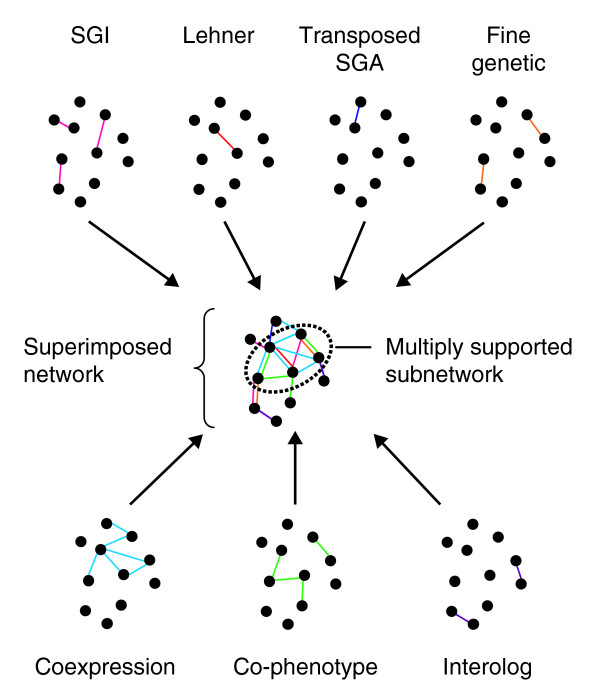
A schematic diagram of the construction of a superimposed network. Networks collected or constructed from various data sources were combined to create the superimposed network. Nodes represent genes; edges are colored according to the data type they represent.

**Figure 7:**
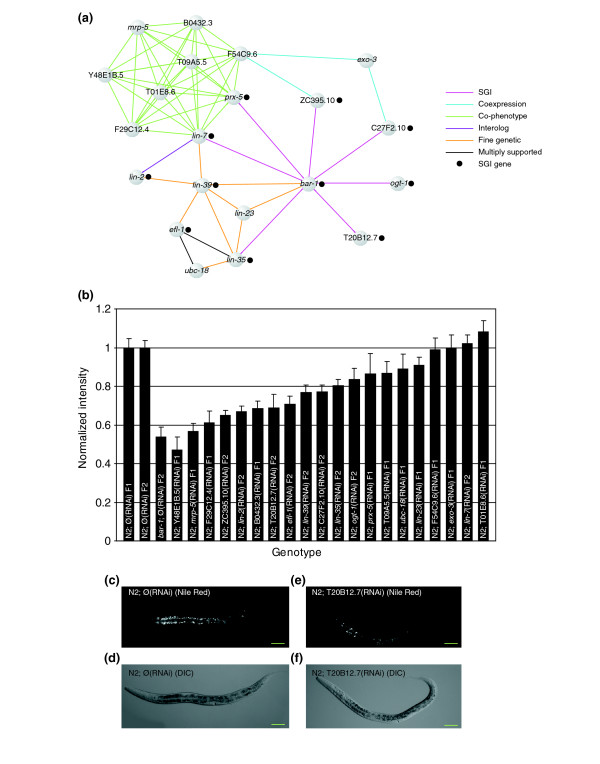
The *bar-1 *module regulates fat storage and/or metabolism. **(a) **The '*bar-1 *module' of 21 genes was identified by virtue of the interconnectedness of coexpression, co-phenotype, genetic, and protein interactions within the superimposed network. Edges are colored according to the type of supporting evidence. Genes tested for interaction with *bar-1 *within the original SGI matrix are indicated (black dot). Visualization generated with Visant [86]. **(b) **Fat accumulation and/or storage disruption in the *bar-1 *module. Genes in the *bar-1 *module were targeted by RNAi in an N2 background. The resulting worms were stained with Nile Red and staining was quantified in order to compare values to N2 worms fed negative control RNAi (see Materials and methods). Fifteen of 20 genes show a reduction of Nile Red staining in an N2 background. Values have been normalized with N2 values for each experiment. Error bars represent standard error of the mean. **(c,e) **Dark-field micrographs of Nile Red staining (shows as bright patches) in N2 worms fed either (c) negative control mock-RNAi (∅ RNAi) or (e) RNAi that targets T20B12.7. **(d,f) **The corresponding differential interference contrast micrographs are shown below the dark-field micrographs. Scale bar, 50 μm.

Next, we examined how SGI interactions contribute to the connectivity of multiply supported subnetworks (MSSNs) within the superimposed network (see Materials and methods). We define MSSNs as highly connected subnetworks of genes composed of qualitatively different data types that do not necessarily overlap (Figure 6). MSSNs may therefore be able to reveal functional modules that emerge from non-overlapping links. Using one approach, we found 68 MSSNs in the superimposed network that may reflect a higher-level organization of gene activity [18], as 82% are significantly enriched for genes with similar functional annotation (see Additional data file 8). Through a second approach (see Materials and methods), we identified an MSSN that we call the '*bar-1 *module', which illustrates how genetic interactions can unite data from disparate sources to reveal coordinate function (Figure 7a). *bar-1 *encodes a β-catenin ortholog that transduces a Wingless signal [34]. The 21 genes of the *bar-1 *module are linked by seven SGI interactions to the *bar-1 *query gene, 11 fine genetic interactions, 36 co-phenotype links, three coexpression links, and one protein-protein interaction link. To further investigate this subnetwork, we targeted all of the genes within the subnetwork with RNAi in a *bar-1*(*ga80*) mutant background. Of the ten gene pairs within the *bar-1 *module that were tested for interaction within the original SGI matrix, nine (90%) retested similarly. An additional seven new genetic interactions were found within the module (Table 4). In total, we found that 12 of the 20 RNAi targets (60%) interacted with *bar-1*(*ga80*), which is three times more than expected compared to *bar-1*(*ga80*) interactions within the SGI matrix (*p *< 10^-4^)^hg^.

**Table 4:** Genetic interactions within the *bar-1 *module

Target gene	*bar-1*-linked (in SGI network)	*bar-1*-linked (retest)
*C27F2.10*	Y	Y
*efl-1*	N	N
*lin-2*	N	N
*lin-7*	Y	Y
*lin-35*	Y	Y
*lin-39*	N	N
*ogt-1*	Y	W
*prx-5*	Y	Y
*T20B12.7*	Y	Y
*ZC395.10*	Y	N
*bar-1*	ND	N
*B0432.3*	ND	Y
*exo-3*	ND	N
*F29C12.4*	ND	Y
*F54C9.6*	ND	Y
*lin-23*	ND	N
*mrp-5*	ND	Y
*T01E8.6*	ND	Y
*T09A5.5*	ND	Y
*ubc-18*	ND	N
*Y48E1B.5*	ND	Y

The target genes are the 21 genes of the *bar-1 *module, including the *bar-1 *query. The second column lists the nine interactions between the targets and the *bar-1 *query within the *bar-1 *module that were tested in the original SGI matrix. Y, an interaction was inferred; N, no interaction was inferred; ND, gene pair not tested in SGI. In the retest, all nodes within the *bar-1 *module were targeted by RNAi in the background of *bar-1*(*gm80*). *ogt-1 *interacted weakly (W) in the direct test, and also had weak interaction scores within the original SGI matrix. We therefore counted *ogt-1 *as a target that behaved similarly in both the SGI matrix and the detailed examination of the *bar-1 *module.

Genes within the *bar-1 *module linked by co-phenotype exhibit a pale and scrawny phenotype when targeted by RNAi [3]. We also found that RNAi-targeted *lin-35 *and *T20B12.7 *exhibit the same pale and scrawny phenotype in a *bar-1*(*ga80*) background. We hypothesized that the pale phenotype is due to decreased fat production or storage. A common method for examining fat accumulation in *C. elegans *is to incubate worms in Nile Red vital dye, which stains lipids and readily accumulates within the triglyceride deposits in the intestine [46]. We therefore targeted each gene within the subnetwork by RNAi in the presence of Nile Red and measured the accumulation of Nile Red microscopically (see Materials and methods). Fifteen of the 20 genes targeted gave a phenotype of significant decrease in Nile Red accumulation in an N2 background (Figure 7b,c). Five of the nine genes that present the pale and scrawny phenotype also showed the decrease in Nile Red staining, suggesting that defects in fat metabolism and/or accumulation may account for the phenotypes observed with the transmitted light dissection microscope. Moreover, 10 of the 11 genes that did not present the pale phenotype also retained less Nile Red than controls. Together, these results suggest that the *bar-1 *module may regulate fat production or storage. Furthermore, the analysis of the *bar-1 *module illustrates how SGI interactions can reveal coordinated activity between otherwise disparate genes within the superimposed network.

### SGI interactions link distinct functional modules

**Figure 8:**
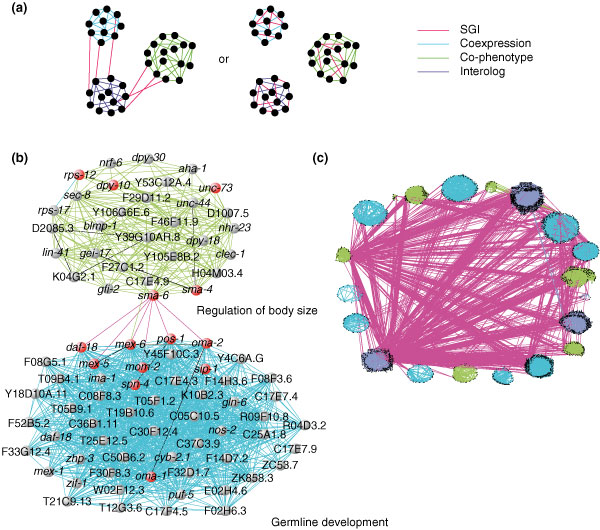
SGI interactions bridge subnetworks. **(a) **Three hypothetical subnetworks are depicted. We asked whether SGI interactions are more likely to bridge subnetworks (left) or fall within subnetworks (right). **(b) **An example of a bridged subnetwork pair is shown. A 'regulation of body size' co-phenotype subnetwork (green links) is linked to a 'formation of primary germline' coexpression subnetwork (blue links) via six SGI interactions (pink links). Visualization generated with Visant [86]. **(c) **Broad subnetworks were identified separately within the coexpression (blue), co-phenotype (green), and interolog (purple) networks (see Materials and methods). All broad subnetworks that are significantly bridged with at least one other broad subnetwork by SGI interactions (pink edges) are shown. Nodes (black dots) represent individual genes. Visualization generated with Visant [86].

The topology of the *bar-1 *module, along with the finding that SGI interactions are largely orthogonal to other types of functional links, raised the possibility that synthetic genetic interactions interconnect, or bridge, functional modules on a global scale. To investigate this possibility, we first identified subnetworks within the coexpression, co-phenotype, and interolog networks that contributed to the superimposed network (see Materials and methods). We found that 162 of the 343 resulting subnetworks (47.2%) are enriched for shared functional annotation (Additional data file 9). We then asked if SGI interactions typically fall within or between subnetworks (Figure 8a). We found 33 subnetwork pairs significantly bridged by SGI links, which is eightfold more than expected by chance (*p *< 10^-23^) (see Materials and methods and Additional data file 10). By contrast, SGI links are significantly under-represented within these subnetworks (*p *< 0.001)^hg^. An example of a pair of subnetworks bridged by SGI interactions is shown in Figure 8b, in which a 'regulation of body size' subnetwork is linked to a 'formation of primary germline' subnetwork, as defined by GO annotation. Interestingly, a 'negative regulation of body size' subnetwork was found to be bridged to the same 'formation of primary germline' subnetwork. Genes within these subnetworks are known to interact with one another in other systems and are discussed below.

To further investigate the propensity of SGI interactions to bridge subnetworks, we relaxed the stringency with which we identified subnetworks to create 'broad' subnetworks that contain up to hundreds of genes (see Materials and methods and Additional data file 9). We reasoned that broad subnetworks are likely to contain genes that belong to common pathways, complexes, and functional modules. Interactions that bridge broad subnetworks are therefore likely to reveal functional redundancy among these components. Consistent with the idea that broad subnetworks are enriched for functional modules, the protein (*p *< 10^-4^)^hg^, coexpression (*p *< 0)^hg^, and co-phenotype (*p *< 10^-26^)^hg ^networks are each significantly enriched for interactions within broad subnetworks (Additional data file 11). By contrast, we found that SGI interactions significantly bridge broad subnetworks (*p *< 10^-6^)^hg ^(Figure 8c). Six hundred and twelve SGI interactions bridge subnetworks, compared to an expected 569.6 based on chance. These results further demonstrate that SGI interactions have the propensity to bridge distinct functional modules. Together, these results provide the first evidence that functional redundancy may extend beyond individual gene pairs to a higher level of organization within the system – the functional module.

### The connectivity of the current-synthetic genetic networks is not conserved between worms and yeast

**Figure 9:**
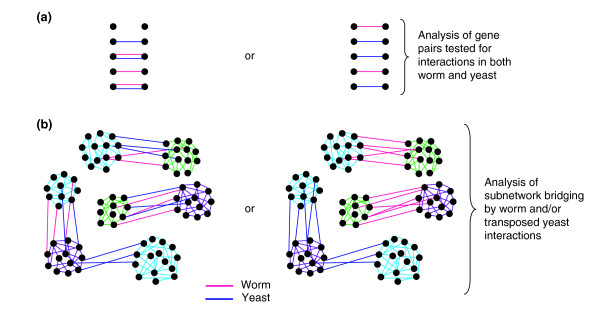
A schematic diagram showing the approaches used to investigate whether synthetic genetic network connectivity is conserved. In all panels, nodes represent genes and lines represent interactions. **(a) **Among pairs of homologous genes tested for interaction in both worm and yeast, we investigated whether there was significant overlap between worm (pink) and yeast (blue) genetic interactions (left), or few overlapping interactions (right). **(b) **After identifying subnetworks (groups of highly interconnected nodes linked by green, purple or light-blue links) within the superimposed network, we investigated whether worm (pink) and yeast (blue) genetic interactions link the same (left) or different (right) subnetworks.

An important question in systems biology is whether genetic-interaction networks are evolutionarily conserved beyond purely network principles. Although only 17% of the gene pairs tested for a genetic interaction in *C. elegans *or *S. cerevisiae *are orthologous, we devised several approaches to investigate whether the connectivity of the current yeast and worm genetic-interaction networks is conserved (Figure 9). First, a direct comparison of SGI interactions and SGA interactions revealed no overlap. As there is very little overlap between the sets of genes tested in both screens, the significance of this result cannot be determined because of a lack of statistical power. Second, we compared a compendium of worm genetic interactions (SGI and Lehner *et al*. [24] genetic interactions) to a compendium of yeast genetic interactions (genetic interactions in BioGrid [41] and SGA interactions [12]). This analysis was restricted to pairs of worm genes tested by SGI and the Lehner study that have yeast homologs. We asked whether genes found to interact in worms were more likely to interact in yeast. Of the gene pairs that interact in worms, 4.7% (2/43) also interact in yeast. However, 4.4% (40/916) of all gene pairs tested in worms also interact in yeast. Thus, an interacting gene pair in *C. elegans *is no more likely than any of the tested gene pairs to interact in *S. cerevisiae *(chi square test, *p *> 0.05). Third, we investigated whether worm and transposed yeast genetic interactions bridge the same subnetworks. For each pair of subnetworks, we determined whether there is a concomitant enrichment of both yeast and worm genetic bridges over what is expected, on the assumption that the worm and yeast datasets are independent of one another (see Materials and methods). We restricted this analysis to pairs of subnetworks such that one subnetwork contains genes that have been tested for interaction with genes in the other subnetwork in both worm and yeast analyses. Of the 274 subnetwork pairs, 27 are significantly bridged by worm links and 35 are bridged by at least one SGA link. Four of these pairs are bridged by both worm genetic interactions and SGA interactions, which is not a significant enrichment (chi square test, *p *> 0.05). Fourth, we repeated the aforementioned analysis using broad subnetworks (see above and Materials and Methods). We found 16 of the 181 possible pairs of broad subnetworks to be bridged by both worm and yeast genetic links, which is not significantly different from the 16.6 pairs expected to be bridged by both types of links by random chance (chi square test, *p *> 0.05). We therefore conclude that the connectivity of the current synthetic genetic-interaction networks is not conserved between yeast and worms.

## Discussion

We developed systematic genetic interaction analysis (SGI) to identify biologically relevant genetic interactions in a systematic and high-throughput manner. Through our unique approach, we were able to extract 3.5-fold more interactions than a previous study [24], despite testing 9.2-fold fewer gene pairs for interaction. The resulting SGI network of 1,246 interactions is the largest metazoan genetic network reported to date. Four lines of evidence support the validity of SGI interactions. First, replicates of 90 query-target pairs were included in both the signaling and the LGIII matrix, yielding a technical reproducibility of 83%. Second, six of the query genes were also included as RNAi targets, yielding a reciprocal reproducibility of 40%. Full reciprocity is not expected because of the varying degree of gene inactivation in the background of different alleles and RNAi conditions. Third, of the 1,165 gene pairs examined in both this study and by Lehner *et al*. [24], SGI identified 64% of the 28 interactions found by Lehner *et al*., and there is 98.9% agreement between the negative calls. Fourth, an independent method of assessing the likelihood of genetic interactions between gene pairs [44] determined that the SGI network is enriched for interactions that are predicted to be true (*p *< 10^-25^).

Four lines of evidence suggest that the interactions uncovered by SGI are also biologically meaningful. First, query genes involved in signal transduction have dramatically more interactions with signaling targets than with random targets. By contrast, a query gene involved in an unrelated process (DNA-damage response) interacts with signaling and random targets with equal frequency. Second, the SGI network contains 26% of all gene pairs within the interaction test matrix that have similar GO annotation, suggesting that our network is greatly enriched for interactions between functionally related genes (*p *< 10^-21^)^hg^. Third, a cluster analysis reveals many expected patterns within the query gene network, and between query and target genes. For example, a *glp-1*-interacting cluster is enriched for 'Notch-receptor processing' activity [47,48], a *sem-5*-interacting cluster is enriched for 'muscle-development' activity [49,50], and a *bar-1 *interacting cluster is enriched for 'establishment of cell polarity' activity. Finally, genetic interactions between genes within the *bar-1 *module predict a common function: the regulation of fat storage or metabolism. Thus, the dataset contains biologically meaningful relationships that can be mined for further insights.

### The SGI approach reveals interactions in an unbiased fashion

The SGI approach facilitates the discovery of interactions with a wide range of strength and reveals many network variants from which the most biologically relevant network can be extracted. Although our chosen SGI network is significantly enriched with known functional categories, a number of criteria can be modified to mine SGI data for more or less stringent interactions. For example, the SGI variant with the most significant precision and recall (see Figure 2a) had greater overlap with predicted interactions than did the larger SGI network (see Figure 4d). With the SGI approach, tailored sets of genetic interactions can be revealed that either facilitate detailed biological analysis by limiting false positives at the expense of some true positives, or facilitate global network analyses by increasing the capture rate of true positives at the expense of including more false positives.

Our chosen SGI network has good recall and precision when compared to other interaction datasets. As a quality benchmark of precision, we considered the network of fine genetic interactions, which is assembled from low-throughput biological analyses and probably contains few false-positive interactions. The SGI network has a precision similar to the network of fine genetic interactions, which suggests that SGI interactions do not simply represent the additive perturbation of functionally unrelated genes. Although much of the precision score of the SGI network is due to interactions among known signaling components, the precision of the LGIII network remains significant, suggesting that more uncharacterized interactions are uncovered within the LGIII network than within the signaling network, as expected.

Surprisingly, the SGI network has a higher recall than all of the other datasets examined. This is not due to the preselection of signaling targets, as a network created with random LGIII targets also has a higher recall than the other datasets. By comparison, the Lehner network [24], which is similar to our signaling network in that it derives from a matrix of preselected signaling genes, has much lower recall than all SGI-related networks. We suspect that the difference lies in the methodology of identifying interactions: The SGI approach detects interactions ranging from weak to strong, while Lehner *et al*. [24] report only strong interactions. Restricting analyses to strong interactions evidently neglects a large proportion of meaningful interactions between genes known to function within the same biological process, and must therefore miss interactions between genes with no previously shared annotation as well.

### The integration of genetic interactions into a superimposed network reveals a new level of organization

To explore how genetic interactions integrate into the biological system, we integrated the SGI interactions with other genetic interactions and with data from the *C. elegans *interactome, transcriptome, and phenome into a superimposed network. An investigation of the overlap between SGI and other contributing interactions within the superimposed network revealed little overlap. Given that only approximately 1% of the links in the superimposed network are multiply supported, this is not surprising. The lack of overlap cannot be attributed solely to the sparseness of available data in the superimposed network, as both the coexpression and co-phenotype networks were created from nearly genome-scale datasets. In addition, the lack of overlap is unlikely to reflect poor-quality data, as we have demonstrated that the interactions within the SGI network and other datasets contain significant numbers of functionally related gene pairs. This paradox may suggest that most high-throughput datasets generated so far have many false negatives. Alternatively, different interaction modes may have little real correspondence with one another, and instead yield complementary information about the system. In either case, a better understanding of biological systems may be achieved by investigating the entirety of superimposed networks and not just multiply supported links.

Three lines of evidence suggest that multiply supported subnetworks can help predict the function of uncharacterized genes. First, the subnetworks are significantly enriched for GO biological processes, suggesting that uncharacterized genes within the subnetworks may have similar functions. Second, a detailed examination of the *bar-1 *module revealed new genetic interactions that were not tested within the SGI matrix. Third, a shared role in fat accumulation was discovered among the genes of the *bar-1 *module. Of note, the gene *prx-5 *of the *bar-1 *module is required for import into peroxisomes, which carry out β-oxidation of long-chain fatty acids, and has previously been identified in a genome-wide screen for fat-regulatory mutants [51,52]. In humans, peroxisomal misregulation results in defective lipid metabolism and is associated with diseases such as Zellweger syndrome [51]. How other components of the *bar-1 *module regulate fat will be an interesting avenue for further investigation. Our data therefore show that the addition of SGI interactions to other datasets enhances the ability to predict gene function.

The general lack of overlap between contributing datasets of the superimposed network, along with the topology of the *bar-1 *module, led us to the finding that SGI interactions bridge different subnetworks. Subnetworks enriched for particular functions probably work towards a common goal and may define a higher level of organization within the cell, such as molecular machines [17] or functional modules [18]. In one example, SGI interactions with *sma-6 *bridge a subnetwork enriched for 'regulation of body size' genes and a subnetwork enriched for 'germline development' genes. SMA-6 is an ortholog of type I TGF-β receptors [53,54]. While *sma-6 *regulates body size, TGF-β signaling can also regulate germline proliferation in both *C. elegans *and *Drosophila *[55-57]. Thus, interactions with *sma-6 *revealed a putative novel redundant function for the two modules. By overlaying SGI interactions onto a superimposed network, we have discovered significant redundancy between functional modules and revealed a new layer of interactions within a biological system.

### The large number of genetic interactions revealed by SGI is not unexpected

Approximately 18% of the 7,008 gene pairs that we tested interact genetically. We rationalize this large fraction of interacting gene pairs uncovered by SGI in four ways. First, genes within the same local neighborhood on a network graph are more likely to interact with each other than with randomly selected targets. For example, in *S. cerevisiae*, 18–24% of genes linked to the same query gene interact with each other, compared to the interaction rate of 1% for the average query [11,12]. Similarly, a majority of the SGI genetic tests are between genes known or predicted to be involved in signal transduction; a relatively high number of interactions may therefore be expected. Second, essential genes genetically interact with more genes than nonessential genes. For example, when conditional alleles of essential yeast genes are used as queries in SGA screens, the fraction of interactions identified is 5.5-fold more than the number of interactions with nonessential queries (0.6%) [13]. Of the 11 query genes investigated in this study, nine are essential. Thus, by using hypomorphic alleles of genes that probably teeter on the brink of collapse, and designing an approach that can reliably detect both strong and weak interactions, we have created a very sensitive system to detect genetic interactions. Third, multicellular organisms may have more vulnerabilities than unicellular organisms. Each cell type within an animal is likely to be governed by a system with a distinct set of genetic vulnerabilities that is different from other cell types. Because compromising the development or physiology of any one of the major tissue types will probably kill the animal, the vulnerability of the entire system is greater than that of any one cell type. This effect may be further compounded by a complex developmental program. Finally, the total number of anticipated genetic interactions in *C. elegans *as revealed by SGI is in the realm of expectation when compared to that of *S. cerevisiae*. On the basis of the fraction of genes that interacted in the LGIII network (14%), which represents a nearly random set of genes, we estimate there to be approximately 61 million genetic interactions in *C. elegans *that involve an essential gene. The number of expected genetic interactions in *C. elegans *as revealed by SGI analysis is therefore around 120 times that of *S. cerevisiae *[11-13]. By comparison, the number of all possible gene pairs in *C. elegans *is around 11-fold more than the number of all gene pairs in *S. cerevisiae*. Thus, the ratio of expected genetic interactions in worms compared to yeast is only around 11-fold more than the respective ratio of all possible gene pairs in both organisms. This difference probably reflects the increase in complexity of nematodes compared to yeast. By contrast, Lehner *et al*. [24] reported an interaction rate of 0.5%. This fraction would suggest that the ratio of the number of expected genetic interactions in worms compared to yeast is around 0.4-fold less than the ratio of all possible gene pairs in worms compared to yeast, which is inconsistent with expectations. We therefore conclude that the number of interactions revealed by SGI is not unexpectedly high.

### The connectivity of synthetic genetic networks may not be evolutionarily conserved

Whether the connectivity of genetic interactions is conserved, rather than just the principles of network biology, remains an open question. A comparison between the only two organisms in which genetic interactions have been systematically investigated – *S. cerevisiae *and *C. elegans *– suggests not. We have evidence against the conservation of genetic interactions at both the level of individual gene pairs and at the level of subnetwork connectivity. Our observations are consistent with a previous report that less than 1% of around 1,000 yeast interactions are conserved in *C. elegans *[58]. How can this be, given that individual genes [59], homologous physical interactions (interologs), the essentiality of hubs, and network principles are all clearly conserved [3,24,37,44,59,60]? There are at least three trivial explanations for the apparent lack of conservation in the connectivity of synthetic genetic networks. First, the different approaches used to uncover interactions may have led to an artificial difference in the genetic network connectivity within the two systems. Second, synthetic genetic-interaction analysis in *C. elegans *has focused on signaling pathways that are largely absent from *S. cerevisiae*, hindering direct comparisons. Third, only a tiny fraction of the synthetic genetic network has been probed in either system. An expanded investigation of the networks may yield more commonalities. Finally, a nontrivial explanation for the apparent lack of conservation may lie in the nature of synthetic genetic networks, which overwhelmingly reveal redundancy between pathways and functional modules as we show here (see also [16,19]). Thus, perturbations in the connectivity between modules may change through random mutation of genes without phenotypic consequence. Over an evolutionary time scale, synthetic genetic relationships may therefore drift and/or be selected for or against to satisfy new constraints during speciation [18,61]. If one mode of evolution is the shuffling of relationships between functional modules, then there may be no reason to expect that the connectivity of genetic networks will be conserved. Whereas model systems have repeatedly proven their utility for discovering and understanding basic biological processes and monogenic diseases, our results suggest that understanding the complex network of interactions that underlie polygenic diseases may require network analysis of systems more closely related to humans. Regardless of this, a study of the connectivity of synthetic genetic networks from different species may provide insight into the evolution of divergent form and function.

## Conclusions

We have developed a novel, sensitive, and reproducible approach called SGI for systematically investigating genetic interactions in *C. elegans*. Using this approach, we identified a network of 1,246 interactions among 461 genes, providing functional annotation for many poorly characterized signal transduction genes. When integrated with other interaction data into a superimposed network, the SGI interactions help reveal new putative functional modules. Because genetic links are largely orthogonal to other interaction modes, SGI data make a significant contribution to connectivity within the superimposed network. Furthermore, SGI interactions link distinct functional modules on a global scale, revealing a new level of organization within the system. Finally, we find that genetic network properties are conserved between yeast and worms, but the connectivity may not be. Together, our results indicate that a comprehensive investigation of genetic interactions is critical to our understanding of the metazoan biological system.

## Materials and methods

### RNAi feeding assay

Query-target gene pairs were tested for interaction by feeding target gene RNAi to worms with a mutation in the query gene. RNAi cultures were grown in 100 μg/ml LB Amp overnight at 37°C. 40 μl of culture was placed on each well of 12-well plates containing 3.5 ml NGM [62] supplemented with 105.6 μg/ml carbenicillin and 1 mM isopropyl-beta-D-thiogalactopyranoside (IPTG). Plates seeded with bacteria were dried overnight at room temperature and for 40 min in a flow hood. Two stage L3–L4 worms (N2, *egl-15*(*n1477*), *let-756*(*s2613*), *sos-1*(*cs41*), *sem-5*(*n2019*), *let-23*(*n1045*), *let-60*(*n2021*), *clk-2*(*mn159*), *daf-2*(*e1370*), *glp-1*(*or178*), *sma-6*(*e1482*), *bar-1*(*ga80*)) were placed in each well of a 12-well plate using a COPAS BioSort worm sorter (Union Biometrica, Holliston, MA). Worms were grown at 20°C (*egl-15*(*n1477*), *let-756*(*s2613*), *sos-1*(*cs41*), *sem-5*(*n2019*), *let-60*(*n2021*), *sma-6*(*e1482*), *bar-1*(*ga80*)) or at 16°C (*glp-1*(*or178*), *let-23*(*n1045*), *clk-2*(*mn159*), *daf-2*(*e1370*)). The following controls were grown in each experiment. As a positive control for RNAi efficiency, wild-type (N2) worms and the query mutants were fed *pop-1*(*RNAi*). As negative controls for background growth levels, N2 worms were fed target RNAi and query mutants were fed L4440 mock-RNAi.

Typically, one person can prepare and process experiments with four worm strains fed 384 RNAi-inducing bacterial strains in triplicate over the course of two weeks. Overlapping sets of experiments of similar size can be prepared while the worms in the first experiment are growing, resulting in an average throughput of 1,920 genetic tests per week per person.

### Analysis of the distribution of functional categories within the LGIII set

Within the LGIII set of genes, there are 203 genes annotated with at least one GO biological process. These genes represent 280 unique GO Process 1000 categories. One thousand samples from the *C. elegans *genome of 203 genes with at least one GO biological process were then chosen randomly. The random set has a mean of 322.5 unique GO Process 1000 categories with a standard of deviation of 32.8. Compared to the random set, there is no significant difference in the number of unique GO processes in the LGIII set (z-score = -1.298; *p *= 0.097 after Bonferroni correction). Furthermore, of the 280 unique GO biological processes in the LGIII set, only 18 are significantly enriched (*p *> 0.01) in the LGIII set, and all of these are represented by only one (12 processes), two (four processes) or three (two processes) genes (see Additional data file 2).

### Scoring query-target interactions

The number of progeny counted in a well that resulted from each query-target pair and control combination was counted and recorded as growth scores. A well with no progeny was given a growth score of zero, whereas a well overgrown with progeny was given a growth score of six. Growth scores of 1 to 5 were assigned to wells with increasing numbers of worms (1, 1–10 progeny; 2, 11–50 progeny; 3, 51–100 progeny; 4, 101–200 progeny; 5, 200+ progeny). From pilot experiments performed by two independent investigators, we found that worm populations can be quickly and reliably binned into these categories. We took several counts of the same maturing population over the course of several days. Each query-target pair and its two controls were tested in at least three rounds. Experiments suspected of contamination were flagged as suspect and repeated. Counts obtained in a round were annotated with confidence scores of 0, 1, or 2, reflecting whether they were suspect, not suspect, or resulted from a second attempt, respectively. A large fraction of all experiments was digitally archived using a high-throughput digital imager [63,64].

### Determination of interactions from growth scores

Let *G*(*Q*, *T*,*i*,*j*) be the growth score for the (*Q*,*T*) query-target pair on the *j*th day of round *i*. For each query-target pair, two growth score differences were calculated: 1, *D*_*null*_(*i*,*j*) = *G*(*Q*,_*null*_,*i*,*j*) - *G*(*Q*,*T*,*i*,*j*), the difference between the experimental population (query mutant; target RNAi) and the mock RNAi vector control (query mutant; L4440 RNAi); and 2, *D*_*wt*_(*i*,*j*) = *G*(*wt*,*T*,*i*,*j*) - *G*(*Q*,*T*,*i*,*j*), the difference between the experimental population and the wild-type control (N2; target RNAi). The following sequential rules were used to call a (*Q*,*T*) pair an interaction:

For round *i*, its *j*th day's counts were called 'deviant' if both *D*_*wt*_(*i*,*j*) and *D*_*null*_(*i*,*j*) were at least *d*.

A round's set of counts was labeled 'positive' if at least *e *of its days were found to be deviant (*e *= 1 or 2) or a majority of its days were deviant (*e *= 0).

A (*Q*,*T*) pair was then called an interaction if at least *s *of its rounds were positive (*s *= 1 or 2) or a majority of its rounds were positive (*s *= 0).

Three additional criteria were used to determine how counts from suspect rounds were treated:

Suspect rounds were excluded from the analysis if the confidence score was less than a threshold *c *(*c *= 0, 1, or 2).

Counts derived from suspect rounds were removed if a second attempt was conducted as long as the parameter *r *was set; if *r *was not set, all counts were retained.

Suspect rounds were included to bring the total number of rounds to a minimum of *m *(*m *= 1 or 2).

### Generation and comparison of network variants

We applied all combinations of the above criteria to generate 51 unique network variants. All interacting pairs within a network variant were query-target pairs that had satisfied all of the criteria imposed by the variant. For example, in a variant with the following criteria: *d *= 3, *e *= 1, *s *= 2, *r *= 1, *c *= 0, and *m *= 2, all query-target pairs that were called interacting were found in at least two (*s *= 2) positive rounds that had at least one deviant day (*e *= 1), for which the difference between the growth scores of the experimental population and the control populations was at least three (*d *= 3). If any round was considered suspect and the experiment for that round had been repeated, only growth scores from the second attempt were used (*r *= 1). Otherwise, rounds with all levels of confidence were used (*c *= 0). If fewer than two rounds of data were available for a specific query-target pair, data from additional rounds were included, so that at least two rounds of data were available, starting from the most confident rounds (*m *= 2).

To compare network variants, we identified pairs of genes within each variant that share a GO biological process classification [26]. Only categories with fewer than 1,000 genes were considered. We calculated 'recall' and 'precision' for each variant, V, as:

Recall (V) = (number of co-classified interacting pairs in V)/(number of possible co-classified pairs) and

Precision (V) = (number of co-classified interacting pairs in V)/(number of interacting pairs in V)

We estimated the significance of the degree to which each network linked genes in the same GO biological process category using the hypergeometric distribution. The hyper-geometric distribution takes into account the number of co-classified interacting pairs in each variant relative to the size of the variant, the total number of all possible co-classified gene pairs, and the total number of gene pairs tested, and is thus a measure of the significance of both the recall and precision of a variant.

### Clustering of interaction strengths

An interaction strength (*IS*) was calculated so that target and query genes could be clustered on the basis of their interaction profiles. The *IS *measures the average difference between the experimental and control populations of worms. For interacting pairs, we averaged *D*_*wt*_(*i*,*j*) and *D*_*null*_(*i*,*j*) using only days and rounds passing criteria 3 to 6. For pairs considered non-interacting, all rounds that passed criteria 4 to 6 were included in the computation. The final interaction strength for a particular query-target pair was calculated as:



where 1(*i*) was 1 if round *i *passed the above criteria and was 0 otherwise, *h *is the total number of rounds that passed the criteria, and *n*_*i *_is the number of days in round *i*. *IS *represents the average growth score for a query-target pair calculated over its valid data.

Target and query genes were clustered on the basis of their interaction strengths. Hierarchical agglomerative clustering was run using Cluster 3.0 [65,66] on both the target and query dimensions using average linkage as the cluster similarity metric and uncentered Pearson correlation as the *IS *profile similarity metric, respectively. Individual target gene clusters were defined by cutting the hierarchical tree at a height of 0.4. The degree to which each cluster contained genes assigned to the same gene functional category was measured using the hypergeometric distribution and a significance cutoff of *P *< 0.01.

### Gene functional categories

We searched for common functional annotation present in clusters of genes generated by the hierachical clustering. To do so, we collected several datasets of gene functional categories described for *C. elegans *genes specifically as well as for predicted *C. elegans *orthologs from other organisms. We collected *C. elegans *gene categories from GO [26] (downloaded from [67] on 17 January, 2007) and KEGG [68] (downloaded from [69] on 13 June, 2005). We restricted GO process categories to those containing 1,000 genes or fewer. Annotations implied by the 'is-a' or 'part-of' subsumption GO hierarchies were automatically added. We also collected *S. cerevisae *gene pathways from MIPS [70] (downloaded on 12 May, 2002) and *H. sapiens *gene pathways from BioCarta [71] (downloaded on 13 June, 2005). For the MIPS and BioCarta datasets, we found the predicted *C. elegans *ortholog for each gene in a pathway by identifying the reciprocal best match protein using the BLASTP program [72]. All of the categories with their associated genes can be found in Additional data file 12.

### Construction of the query network

Pairs of query genes found to interact with a significantly similar set of target genes were connected by 'congruent links' as defined by Tong *et al*. [12] and Ye *et al*. [32]. The *P *value of the overlap of *k *target genes of a query gene pair (A,B) was determined using the hypergeometic distribution:



where *K *is the number of target genes linked to query gene A, *n *is the number of target genes linked to query gene B, and *N *is the number of tested target genes. A *P *value cutoff of *p *< 10^-9 ^yielded a total of 16 congruent links.

### Testing the correlation of target hubs with RNAi phenotype

We tested whether targets with high degree (those linked to many query genes) have an increased tendency to produce a strong phenotype when targeted by RNAi compared to targets with low degree (those linked to few query genes). The phenotype data of Kamath *et al*. [3] were used. We define a strong phenotype as any of the following: Emb (embryonic lethal), Ste (sterile), Let (lethal), Lva (larval arrest), Lvl (larval lethal), or Adl (adult lethal). Our null hypothesis is that the degree of a target gene is not correlated with strong RNAi phenotypes. Under the null hypothesis, we expect to find an equal proportion of strong RNAi phenotypes among targets with any degree. We quantified the difference between the observed and expected number of target genes with a strong RNAi phenotype for each degree using a chi-square test with 10 degrees of freedom (one less than the number of query genes).

### Comparing the network properties of the SGI and SGA genetic networks

To measure topological network properties of the SGI and yeast SGA genetic-interaction networks, we used the program tYNA [73] to analyze the variance of the SGI and yeast SGA network properties. The resulting standard errors of the mean for the SGI network parameters are reported in the text.

### Construction of the co-phenotype network

A co-phenotype network was created by linking genes with similar loss-of-function phenotypes detected in recently published high-throughput RNAi screens [3,4,42]. An RNAi phenotype compendium was assembled by compiling the results of three genome-wide RNAi studies: 31 phenotypes scored for 1,472 RNAi from the Kamath *et al*. [3] dataset; 25 phenotypes scored for 1,486 RNAi from the Simmer *et al*. [4] dataset; and 26 phenotypes scored for 1,066 RNAi from the Rual *et al*. [42] dataset. Several phenotypic annotations in the datasets were converted to provide a uniform terminology that allowed the three datasets to be integrated. These conversions included labeling brood counts scored as '1–5' and '6–10' as 'Ste'; relabeling 'Prz' as 'Prl'; relabeling 'Lvl' as 'Let'; and labeling any embryonic lethal percentages over 10% as 'Emb.' In total, 37 phenotypes scored across 2,327 unique RNAi experiments were collected from the three studies and recorded in a 2,327 × 37 RNAi phenotype matrix, *K*. Each entry in the matrix, *K*_*iv *_was set to 1 if RNAi against gene *i *produced phenotype *v *in one of the three studies and was set to 0 otherwise. Each row in the matrix is referred to as a gene's RNAi phenotype profile.

We devised a measure of phenotypic similarity motivated by the uncentered Pearson correlation coefficient (phenotypic PCC) approach of Gunsalus *et al*. [39]. However, we chose not to use the phenotypic PCC as it can produce false-positive links between genes with a high correlation that is based on a single (or even a few) shared common phenotype(s) when the two genes fail to produce phenotypes in all (or many) of the other phenotypes. Inspection of the compiled RNAi phenotype dataset reveals thousands of gene pairs that result in such spurious, yet perfect, correlation. In addition, phenotypic PCC will result in false negatives due to low correlations between genes that share several rare phenotypes but that differ in only a few others.

We suggest that a good measure of similarity should give more weight to rare phenotypes as opposed to common phenotypes shared between genes because infrequent phenotypes will co-occur less often in two genes by chance. Furthermore, the similarity between two genes should increase if both do not produce a very common phenotype when genes are targeted by RNAi. We calculated a loss-of-function agreement score, LOFA, for two genes *i *and *j*, that captures these intuitions, defined as:



where *f*_*v *_is the frequency of phenotype *v *across the genome and *K*_*iv *_is the (*i,v*)th entry from the RNAi phenotype compendium matrix as described above. If RNAi produces phenotype *v *in two genes, the LOFA score is increased by -log(*f*_*v*_). The boost is larger for more infrequent phenotypes. For example, a phenotype that occurs in 1 out of 100 genes will increase the score by 2 units, whereas a phenotype that occurs in 1 out of 10 genes will contribute only 1 unit of score. The LOFA's second term gives a bonus to two genes if they both do not share a common phenotype in an analogous fashion.

The LOFA and phenotypic PCC measures of similarity were compared by measuring their ability to predict genes of related function. For each score, we constructed networks induced by using a cutoff above which genes were considered to be functionally related. We first varied the LOFA score cutoff from high to low, producing 51 networks of increasing size. Similarly, 51 networks of increasing size were produced for phenotypic PCC by lowering the phenotypic PCC cutoff. The precision of each network was measured by calculating the fraction of linked genes found to be annotated with a common GO category. Precision levels were then plotted against the network size. LOFA was found to be superior to phenotypic PCC for connecting genes of related function as it produced substantially higher precision levels than phenotypic PCC for every network size (Additional data file 13).

A final co-phenotype network was constructed by linking genes exhibiting significant levels of agreement. The significance of the LOFA score was assessed by generating 3 million random LOFA values. We first constructed a random dataset in which the genes associated with loss-of-function phenotype *v *in the RNAi phenotype compendium were permuted. This was repeated for each phenotype to produce one permuted dataset from which 100,000 random pairs were then picked and LOFA was calculated. We repeated this procedure for 30 different permuted datasets. We found that a cutoff of 7.0 was equivalent to an estimated significance level of 0.001, as approximately 100 LOFAs computed from random datasets exceeded this value on average in each of the 30 permuted trials.

### Construction of the transposed SGA network and the interolog network

We constructed the transposed SGA network of synthetic genetic interactions from those interactions described in [12] by mapping each yeast gene to its predicted worm ortholog(s). Maps were created containing all gene pairs with BLASTP significance values of *p *< 10^-30 ^or better [72]. For interactions between yeast genes with multiple predicted worm orthologs, transposed interactions were created for all combinations of predicted orthologs.

The interolog network was created from eukaryotic protein-protein interactions reported in BioGRID [41]. All interactions assembled from organisms other than *C. elegans *were mapped to predicted worm ortholog pairs using BLASTP with a significance cutoff of *p *< 10^-30 ^[72].

### Construction of permuted networks

To gauge the significance of various network properties, 1,000 randomly permuted networks were constructed for each data type. Permuted SGI networks were created by combining permuted signaling and LGIII networks. A link in each of these networks associates one query gene with one RNAi target gene. The permuted SGI networks link each query gene to a random set of target genes by randomly picking genes from the entire set of target genes tested in the screen. The number of target genes linked to each query was held fixed in the permuted networks to preserve the degree distribution across query genes. We also created permuted Lehner *et al*. [24] networks, yeast SGA networks, and protein-interaction networks using this method. Permuted coexpression, co-phenotype, and fine genetic networks were created by randomly linking genes present in each network. Random superimposed networks were created by taking the union of all links from the permuted networks obtained from the separate data types.

### Determination of the significance of the number of supported links

The significance of the number of supported links (gene pairs linked by more than one data type) in the superimposed network was estimated by comparing the observed number of supported links to the number of supported links in 1,000 randomly permuted superimposed networks. Significance was calculated with a standard Z-score transformation using the mean and standard deviation of the number of supported links across the random networks. The significance of the overlap of two data types was estimated in a similar manner.

### Identification of gene subnetworks

We identified subnetworks, defined as small- to medium-sized groups of possibly overlapping genes, by searching for densely connected sets of genes in individual networks and in the superimposed network using MODES [74]. We used MODES parameter settings such that a subnetwork must have at least 50% connectivity, cannot overlap any other subnetwork by more than half of its genes, and must contain a minimum of four genes.

A connectivity significance score was assigned to each subnetwork based on the number of links connecting each of its members. The connectivity significance score for a subnetwork containing *n *genes was calculated as a standard Z-score (*l *- *m*)/*s *where *l *is the observed number of links in the subnetwork, and *m *and *s *are the mean and standard deviation of the number of links across 1,000 random collections of *n *genes.

As a post-processing step, any gene that was not grouped into a subnetwork by MODES was iteratively considered for addition to each subnetwork. To achieve this, a hierarchical clustering merge step was performed on all such genes across all subnetworks, using the connectivity score as the basis for a similarity metric. At each step in the clustering, the gene/subnetwork pair with the largest increase in connectivity score was combined. The connectivity score increase was calculated as the subnetwork's connectivity score upon addition of the gene minus its connectivity score before the addition of the gene.

Broad subnetworks were identified in single-data-type networks using the VxOrd algorithm [40]. VxOrd clusters a network of genes on a two-dimensional surface using multidimensional scaling [75]. The links between genes are treated as spring constants and a configuration of the springs is sought that minimizes the total free energy of the system. The result is a collection of genes arranged on the *X*-*Y *plane. We partitioned the genes into clusters using the dense subregions obtained from two-dimensional density estimation over a grid superimposed on the *X*-*Y *plane. We formed clusters of genes in contiguous regions whose densities were at least 10% of the maximum density and matched a minimum area cutoff.

### Characterization of multiply supported subnetworks

Each subnetwork identified in the superimposed network was inspected to determine which types of data significantly link its gene members. For each subnetwork, the significance of the number of links of a specific data type that connected two genes within the subnetwork was calculated using the connectivity significance score (see previous section). Subnetworks were annotated as enriched for a data source if the connectivity score had an associated *P *value of 0.01 or less.

The *bar-1 *module was identified in a search for multiply-supported subnetworks within an earlier version of the superimposed network. The links within the subnetwork were updated using the same data as reported in the current subnetwork. This resulted in the addition of two links to the module: an interolog interaction between *efl-1 *and *lin-35 *and a Lehner interaction between *ubc-18 *and *lin-35*.

### Nile Red analysis

L4 parental worms were placed on NGM plates seeded with RNAi or mock-RNAi bacteria and 0.015 μg/ml Nile Red. L4 F1 and F2 progeny were analyzed by fluorescence microscopy for Nile Red intensity. To quantify Nile Red intensity, Openlab software (Improvision, Lexington, MA) was used to calculate mean fluorescence within a measured area as well as the length of the worm. Nile Red intensity was calculated as: mean fluorescence × area/length of worm.

### Identification of significantly bridged subnetwork pairs

All pairs of subnetworks derived from the coexpression, co-phenotype, and interolog networks were inspected for significant bridging by SGI links. An SGI link is considered to bridge a pair of subnetworks if it connects a gene in one subnetwork to a gene in another subnetwork. The total number of bridges was counted for each pair of subnetworks. The significance of the number of bridges for each subnetwork pair was then determined with a standard Z-score transformation using the mean and standard deviation of the number of bridges between that subnetwork pair in 1,000 randomly permuted SGI networks (see Additional data file 14 for evidence that a normal approximation in the Z-score transformation is valid). In addition to a cutoff of *P *< 0.01, a subnetwork pair was required to have at least three bridges to be considered significantly bridged.

### Estimation of the significance of the number of bridged subnetwork pairs

We estimated the significance of the number of significantly bridged subnetwork pairs by comparing to the number of pairs significantly bridged by permuted SGI networks. Each of the 1,000 randomly permuted SGI networks was used to search for significantly bridged subnetwork pairs using the same method described above for the true SGI network. The mean and standard deviation of the number of significantly bridged subnetwork pairs were then calculated across all permuted networks. The number of subnetwork pairs significantly bridged by the SGI network was then compared to these values using a standard Z-score transformation to obtain a single significance value.

### Determination of bridging propensities

To measure the propensity for a given data type to bridge subnetworks more than expected by chance, we restricted our analysis to all subnetwork-to-subnetwork links (SSLs). We defined an SSL as a linked gene pair (A,B) in which both A and B were included in at least one broad subnetwork of any data type. Over all SSLs we counted the number of 'supports', those links in which genes A and B occurred in the same subnetwork, as well as 'bridges', those links in which A and B occurred in separate subnetworks. Links that both bridge and support were counted as supports. The 'bridging fraction' was then calculated as the total number of bridges divided by the total number of SSLs. The observed bridging fraction was calculated using all SSLs in the network. The expected bridging fraction was calculated using all SSLs tested in the dataset. To measure the tendency for a given data type to link across versus within broad subnetworks, we calculated the 'bridging propensity' as the observed bridging fraction divided by the expected bridging fraction, minus 1. Positive bridging propensities are indicative of a link type tending to bridge (as opposed to fall within) broad subnetworks more than expected by chance.

### Determination of the degree of subnetwork bridging conservation

To determine if the same subnetwork pairs were bridged in worm and yeast, we identified significantly bridged subnetwork pairs separately in each species. We used a compendium of SGI and Lehner *et al*. [24] interactions for worm, and transposed SGA links for yeast. We examined all pairs of subnetworks and broad subnetworks separately. We calculated the expected number of bridges as the number of possible (tested) gene pairs between the subnetworks times the probability of linking a gene pair for that data type. An estimate of the probability of a data type linking a gene pair was calculated as the number of links in its network divided by the number of possible (tested) links. This yielded an estimated background probability of 0.039 for worm, and 0.034 for yeast.

To determine the degree of subnetwork bridging conservation among all possible pairs of subnetworks, we created contingency tables containing the observed and expected number of subnetwork pairs significantly bridged only in worm, only in yeast, in both, and in neither. The expected number of pairs for each of these four categories was then calculated, assuming independence of worm and yeast bridging. We first calculated the worm bridging probability, *P*_*w *_(*P*_y _for yeast), as the number of bridged subnetwork pairs divided by the total number of pairs, *N*. The expected number of subnetwork pairs bridged only in worm was then calculated as *NP*_*w *_(1 - *P*_*y*_). Likewise, the expected number of bridged pairs only in yeast was calculated as *N *(1 - *P*_*w*_) *P*_*y*_. The expected number of bridged pairs in both species was calculated as *NP*_*w *_*P*_*y*_. Finally, the expected number of pairs bridged by neither was *N *(1 - *P*_*w*_)(1 - *P*_*y*_). We used a chi-square test with 3 degrees of freedom to determine if the observed and expected counts for each of these categories were significantly different.

## Additional data files

Additional data file 1 is a table listing average growth scores for each query-target pair tested in the SGI analysis. Additional data file 2 is a table listing the distribution of functional categories within the LGIII set. Additional data file 3 is a table listing gene interactions in networks created for this study. Additional data file 4 is a table with a sorted list of average interaction strengths for each query-target pair tested. Additional data file 5 contains a detailed assessment of the nature of the SGI interactions. Additional data file 6 is a table listing reciprocal query-query interactions. Additional data file 7 is a clustered table of growth scores. Gene function descriptions are from WormBase version 170 [43]. Additional data file 8 is a table listing multiply supported subnetworks enriched for genes with similar GO annotations. Additional data file 9 is a table listing genes and functional annotations for all subnetworks. Additional data file 10 is a table listing 33 focused subnetwork pairs along with the corresponding enrichment of SGI links that bridge them. Additional data file 11 is a table comparing bridging propensities among high-throughput datasets. Additional data file 12 is a table listing all functional categories and their associated genes. Additional data file 13 is a figure plotting precision levels of networks created using various cutoffs of the LOFA and PCC scores against network size. All files are also accessible at [76]. Additional data file 14 presents evidence supporting the validity of using normal approximation of the Z-transformation to estimate bridging significance.

## Supplementary Material

Additional data file 1 A table presenting average growth scores for each query-target pair tested in the SGI analysis. Enhancing (E) and alleviating (A) interactions are also indicated. Click here for file

Additional data file 2 LGIII target genes were analyzed for enrichment of specific GO annotation. Click here for file

Additional data file 3 Gene interactions in the SGI, query, and superimposed networks. Click here for file

Additional data file 4 Average interaction strengths for each query-target pair tested are listed. Click here for file

Additional data file 5 A detailed assessment of the nature of the SGI interactions. Click here for file

Additional data file 6 Hypomorphic query worms (x-axis) were fed RNAi that targets query genes (y-axis) to measure the reciprocity of SGI. Average growth scores are indicated for each query (mutant); query (RNAi) interaction (E). Click here for file

Additional data file 7 Targets are included in the table if they interacted with at least one query. Genes are clustered in two dimensions based on the calculated average growth scores. Clusters are labeled A-L and significant enrichment of functional annotation among the genes in each cluster is indicated where applicable (see Materials and methods). Gene function descriptions are from WormBase version 170 [43]. Click here for file

Additional data file 8 MSSNs are listed along with the contributing datasets that make up each MSSN. The amount and significance of GO enrichment among genes of the MSSNs are also indicated. Click here for file

Additional data file 9 Genes and functional annotations for all subnetworks. Click here for file

Additional data file 10 33 focused subnetwork pairs are listed along with the corresponding enrichment of SGI links that bridge them. Click here for file

Additional data file 11 The bridging propensity of various data types is represented. Click here for file

Additional data file 12 All functional categories and their associated genes are listed. Click here for file

Additional data file 13 Precision levels of networks created using various cutoffs of the LOFA and PCC scores are plotted against network size. The arrow indicates the chosen co-phenotype network variant. Click here for file

Additional data file 14 Evidence supporting the validity of using a normal approximation in the Z-transformation to estimate bridging significance. Click here for file
