# Supplementary material for: A global analysis of genetic interactions in Caenorhabditis elegans
Source: J Biol. 2007 Sep 26;6(3):8. doi: 10.1186/jbiol58 (PMC2373897; doi:10.1186/jbiol58)
Supplement: Additional data file 5 — A detailed assessment of the nature of the SGI interactions. [file jbiol58-S5.doc]

**Additional File 5. The SGI interactions are synthetic.**

We developed an approach to determine the network of genetic interactions with the best sensitivity and precision among 52 variant networks. Among the various criteria of the chosen “SGI network” is that each gene pair must result in a score of at least 2 less than either single gene-perturbation control. Calculations using our semi-quantitative scoring scheme show that the measured phenotype of all of the interacting gene pairs exceeds the product of the measured phenotype of the single gene-perturbation controls and are therefore *bona fide* synthetic genetic interactions. Two examples are shown below:

Example 1:

|  | **A** | **B** | **A+B**  **observed** | **A+B**  **expected** |
| --- | --- | --- | --- | --- |
| **Method A** |  |  |  |  |
| Growth Score | 5 | 3 | 1 | 2 or 3^d^ |
| Fraction of WT (X/6)^a^ | 0.83 | 0.5 | 0.17 | 0.42 |
|  |  |  |  |  |
| **Method B** |  |  |  |  |
| # of Worms (μ)^b^ | 200 | 75 | 6 | 60^e^ |
| Fraction of WT (X/250)^c^ | 0.8 | 0.3 | 0.024 | 0.24 |

Example 2:

|  | **C** | **D** | **C+D**  **observed** | **C+D**  **expected** |
| --- | --- | --- | --- | --- |
| **Method A** |  |  |  |  |
| Growth Score | 4 | 4 | 2 | 3^d^ |
| Fraction of WT (X/6)^a^ | 0.66 | 0.66 | 0.33 | 0.44 |
|  |  |  |  |  |
| **Method B** |  |  |  |  |
| # of Worms (μ)^b^ | 150 | 150 | 30 | 90^e^ |
| Fraction of WT (X/250)^c^ | 0.6 | 0.6 | 0.12 | 0.36 |

Note: Both examples are hypothetical

^a^Shown is the experimental growth score divided by the known wild-type growth score (i.e. 6).

^b^Shown is the average number of worms for that bin. For example, a score of 4 represents between 100 and 200 worms, the average of which is 150.

^c^Shown is the average number of worms for that bin for that experiment divided by the approximate number of wild-type worms in control wells (i.e. 250).

^d^The expected growth score is calculated from the expected fraction of wild-type worms multiplied by 6, the expected growth score of wild-type worms. In example 1, this is 0.42 X 6 =2.5 (i.e. a growth score of 2 or 3).

^e^The expected number of worms is calculated from the expected fraction of wild-type worms multiplied by 250, the expected number of wild-type worms in control wells. In example 1, this is 0.36 X 250 =90.

**A more detailed assessment of the nature of the SGI interactions.**

Our method for determining a genetic interaction, as outlined in the accompanying Byrne et al. manuscript, identifies interactions that exceed phenotypic expectations for a synthetic genetic interaction based on multiplicative models (see Segre *et al*. 2005. *Nature Genetics* 37, 77-83; Schuldiner *et al*., 2005. *Cell* 123, 507) as illustrated below.

The multiplicative model corresponds to calling an interaction whenever the following inequality is true:

$1<\frac{F_{Q0}F_{0T}}{F_{QT}}$,

where *F_QT_* is a measure of fitness for the *Q-T* mutant. *F_Q0_* and *F_0T_* denote the fitness of the query RNAi in wild-type background and the control vector in the hypomorph background respectively. The numerator is the product of the two fitness measures and is interpreted as the expected fitness under the model that genes *Q* and *T* do not interact. If we use the proportion of counted worms as the fitness measure (i.e. *F_QT_ = N_QT_ / W*, where *N_QT_* is the number of worms counted for the *Q-T* pair and *W* is the total possible number of worms) we get the following multiplicative rule:

.

In our case, *W*=250 is assumed as worms were often found burrowing at counts this high, indicating the nutrients in the medium were depleted when the population reached approximately 250 worms.

In contrast, the rule described in the manuscript calls an interaction whenever the following inequality holds:

,

where *d* is the difference cut-off as discussed in the manuscript, and *G_QT_* is the growth score of the *Q-T* query-target double-mutant. While this rule is additive in the growth scores, it is multiplicative in the original worm counts on a plate because our discretization of growth scores is roughly logarithmic (log base 3) with respect to the counts. Thus, the above rule can be rewritten as:

,

where *N_QT_* corresponds to the approximate count of worms for the Q-T mutant.

Using the definitions of the two rules, we see that the interaction calling method used in the manuscript will be more conservative than the multiplicative rule whenever the ratio between the minimum and the product of the control counts obeys the following relation:

.

Since *min(x,y)/xy* is maximum when *x=y* for non-negative integers *x* and *y*, we see that our method is conservative with respect to the multiplicative model (i.e. will call a subset of the interactions as the multiplicative model) as long as the number of worms of the controls are greater than 25. Because this corresponds to a growth score of 2 and our delta parameter was equal to 2, all of the calls reported in the manuscript fall into this conservative range.
