# Supplementary material for: A global analysis of genetic interactions in Caenorhabditis elegans
Source: J Biol. 2007 Sep 26;6(3):8. doi: 10.1186/jbiol58 (PMC2373897; doi:10.1186/jbiol58)
Supplement: Additional data file 6 — Hypomorphic query worms (x-axis) were fed RNAi that targets query genes (y-axis) to measure the reciprocity of SGI. Average growth scores are indicated for each query (mutant); query (RNAi) interaction (E). [file jbiol58-S6.doc]

|  |  | **bar-1** | **egl-15** | **let-60** | **let-756** | **sem-5** | **sos-1** |
| --- | --- | --- | --- | --- | --- | --- | --- |
| **RNAi** | **Gene** | *C54D1.6* | *F58A3.2* | *ZK792.6* | *C05D11.4* | *C14F5.5* | *T28F12.3* |
| **C54D1.6** | *bar-1* | N/A |  | 5.2500 (E) | 4.5625 (E) | 3.4231 (E) | 5.3333 (E) |
| **F58A3.2** | *egl-15* | 1.5000 (E) | N/A |  | 5.1250 (E) | 3.5000 (E) | 2.0000 (E) |
| **ZK792.6** | *let-60* | 1.6667 (E) | 5.1667 (E) | N/A | 4.6364 (E) | 5.1667 (E) | 3.4286 (E) |
| **C05D11.4** | *let-756* |  |  | 1.5000 (E) | N/A |  |  |
| **C14F5.5** | *sem-5* |  | 4.0000 (E) | 4.7500 (E) | 4.7500 (E) | N/A | 2.7500 (E) |
| **T28F12.3** | *sos-1* | 2.1667 (E) |  |  | 1.9444 (E) | 3.0000 (E) | N/A |

**Additional Data File 6. Reciprocal Interactions**
