# Supplementary material for: A global analysis of genetic interactions in Caenorhabditis elegans
Source: J Biol. 2007 Sep 26;6(3):8. doi: 10.1186/jbiol58 (PMC2373897; doi:10.1186/jbiol58)
Supplement: Additional data file 8 — MSSNs are listed along with the contributing datasets that make up each MSSN. The amount and significance of GO enrichment among genes of the MSSNs are also indicated. [file jbiol58-S8.doc]

|  |  |  |  | **Module** | | **Genome** | |
| --- | --- | --- | --- | --- | --- | --- | --- |
| **Subnetwork ID** | **SGI,**  **Fine Genetic,**  **Co-phenotype,**  **Interolog,**  **Co-expression, Transposed SGA** | **Top Enriched**  **Go Category** | **Significance (log)** | **# genes with that annotation** | **# annotated genes** | **#genes with that annotation** | **# annotated genes** |
| Module59_71_784 | 0,0,1,1,1,0 | protein biosynthesis | -47.46 | 49 | 56 | 150 | 1735 |
| Module73_79_784 | 0,0,1,1,0,0 | morphogenesis of an epithelium | -46.19 | 55 | 74 | 150 | 1735 |
| Module86_59_912 | 0,0,0,1,1,1 | protein amino acid phosphorylation | -29.48 | 34 | 47 | 129 | 1735 |
| Module89_57_422 | 0,0,1,1,1,0 | protein catabolism | -25.55 | 18 | 33 | 30 | 1735 |
| Module40_44_244 | 0,1,1,0,0,0 | morphogenesis | -22.80 | 41 | 43 | 429 | 1735 |
| Module41_45_257 | 0,0,1,1,1,0 | determination of adult life span | -21.62 | 22 | 43 | 63 | 1735 |
| Module99_68_584 | 0,1,1,0,1,0 | positive regulation of body size | -18.54 | 25 | 32 | 188 | 1735 |
| Module35_33_136 | 0,0,0,1,1,0 | regulation of protein activity\epigenetic | -18.39 | 13 | 17 | 43 | 1735 |
| Module83_40_199 | 0,0,0,1,1,1 | cytoskeleton organization and biogenesis | -15.46 | 14 | 25 | 53 | 1735 |
| Module54_44_242 | 0,0,1,1,1,0 | generation of precursor metabolites and energy | -14.73 | 18 | 29 | 112 | 1735 |
| Module43_30_122 | 0,0,1,0,0,1 | small GTPase mediated signal transduction | -14.65 | 14 | 29 | 50 | 1735 |
| Module42_29_105 | 1,0,1,0,1,0 | molting cycle (sensu Protostomia and Nematoda) | -14.24 | 13 | 26 | 47 | 1735 |
| Module37_33_150 | 0,0,1,0,1,0 | locomotion | -13.60 | 20 | 32 | 154 | 1735 |
| Module23_13_37 | 0,0,1,0,1,0 | chromosome segregation | -12.87 | 8 | 9 | 36 | 1735 |
| Module77_39_190 | 0,0,0,1,1,0 | DNA replication initiation | -12.66 | 7 | 30 | 7 | 1735 |
| Module82_21_78 | 0,0,1,0,0,1 | small GTPase mediated signal transduction | -12.38 | 10 | 15 | 50 | 1735 |
| Module58_27_103 | 0,0,1,1,1,0 | protein biosynthesis | -12.21 | 15 | 20 | 150 | 1735 |
| Module57_40_199 | 0,0,1,1,1,0 | protein biosynthesis | -10.20 | 18 | 36 | 150 | 1735 |
| Module69_29_105 | 0,0,0,1,1,0 | transcription | -9.44 | 14 | 25 | 136 | 1735 |
| Module34_44_244 | 0,0,0,1,1,0 | proteolysis | -9.32 | 10 | 16 | 89 | 1735 |
| Module71_21_54 | 0,0,0,1,1,0 | transcription initiation | -8.87 | 5 | 18 | 7 | 1735 |
| Module53_21_54 | 0,0,0,1,1,0 | glycolysis | -7.99 | 5 | 15 | 11 | 1735 |
| Module45_19_50 | 0,1,1,0,0,0 | oviposition | -7.69 | 9 | 15 | 101 | 1735 |
| Module47_22_63 | 1,1,0,0,0,0 | cell fate specification | -7.67 | 5 | 17 | 11 | 1735 |
| Module15_14_23 | 0,1,0,1,0,0 | cell death | -7.42 | 5 | 10 | 21 | 1735 |
| Module64_47_309 | 0,0,1,1,1,0 | gametogenesis | -6.96 | 19 | 30 | 334 | 1735 |
| Module39_45_258 | 0,0,1,1,0,0 | osmoregulation | -6.21 | 13 | 37 | 125 | 1735 |
| Module16_15_30 | 1,1,0,0,0,0 | gastrulation | -5.56 | 5 | 14 | 32 | 1735 |
| Module50_6_8 | 0,1,1,0,0,0 | oviposition | -5.46 | 5 | 6 | 101 | 1735 |
| Module136_13_21 | 0,0,0,1,1,0 | protein folding | -5.32 | 3 | 7 | 10 | 1735 |
| Module65_27_90 | 0,0,0,1,1,0 | RNA processing | -4.86 | 4 | 16 | 18 | 1735 |
| Module60_30_111 | 0,0,1,1,1,0 | translation | -4.82 | 6 | 20 | 52 | 1735 |
| Module28_19_44 | 0,0,1,0,1,0 | osmoregulation | -4.47 | 7 | 15 | 125 | 1735 |
| Module49_13_27 | 0,1,0,1,0,0 | monovalent inorganic cation transport | -4.10 | 3 | 5 | 36 | 1735 |
| Module67_21_54 | 0,0,1,1,1,0 | phenylalanyl-tRNA aminoacylation | -4.04 | 2 | 17 | 2 | 1735 |
| Module72_15_32 | 0,1,0,1,1,0 | response to oxidative stress | -3.88 | 2 | 12 | 3 | 1735 |
| Module90_29_108 | 0,1,0,1,0,1 | protein amino acid phosphorylation | -3.70 | 8 | 24 | 129 | 1735 |
| Module46_22_61 | 1,0,0,1,0,0 | signal transduction | -3.48 | 7 | 17 | 153 | 1735 |
| Module52_19_43 | 0,0,0,1,1,0 | protein transport | -3.48 | 4 | 11 | 59 | 1735 |
| Module24_7_13 | 0,0,1,0,1,0 | nucleosome assembly | -3.44 | 2 | 5 | 11 | 1735 |
| Module76_12_18 | 0,0,0,1,1,0 | DNA metabolism | -3.23 | 3 | 5 | 70 | 1735 |
| Module18_54_364 | 0,0,0,1,1,0 | chromosome segregation | -3.06 | 4 | 22 | 36 | 1735 |
| Module108_11_15 | 0,0,0,1,1,0 | ubiquitin cycle | -2.97 | 2 | 7 | 13 | 1735 |
| Module79_22_61 | 0,0,0,1,1,0 | secretion | -2.74 | 2 | 13 | 9 | 1735 |
| Module21_62_475 | 0,1,0,0,1,0 | sexual reproduction | -2.72 | 11 | 23 | 339 | 1735 |
| Module94_16_31 | 0,1,0,1,1,0 | programmed cell death | -2.56 | 2 | 9 | 16 | 1735 |
| Module63_32_131 | 0,0,0,1,1,0 | gametogenesis | -2.50 | 8 | 15 | 334 | 1735 |
| Module95_15_29 | 0,0,0,1,1,0 | carbohydrate metabolism | -2.49 | 3 | 10 | 56 | 1735 |
| Module87_16_31 | 0,0,0,1,1,0 | proteolysis during cellular protein catabolism | -2.19 | 2 | 11 | 20 | 1735 |
| Module62_19_45 | 0,0,1,0,1,0 | gametogenesis | -2.03 | 6 | 11 | 334 | 1735 |
| Module61_12_24 | 0,0,1,0,1,0 |  |  |  |  |  |  |
| Module98_16_33 | 0,1,0,0,1,0 |  |  |  |  |  |  |
| Module74_19_45 | 0,0,0,1,1,0 |  |  |  |  |  |  |
| Module70_25_76 | 0,0,0,1,1,0 |  |  |  |  |  |  |
| Module66_28_95 | 0,0,0,1,1,0 |  |  |  |  |  |  |
| Module32_29_102 | 0,0,0,1,1,0 |  |  |  |  |  |  |
| Module159_4_5 | 0,1,0,1,0,0 |  |  |  |  |  |  |
| Module150_6_6 | 0,0,1,0,1,0 |  |  |  |  |  |  |
| Module123_10_13 | 0,0,0,1,1,0 |  |  |  |  |  |  |
| Module117_6_7 | 0,0,0,1,1,0 |  |  |  |  |  |  |
| Module109_7_8 | 0,0,0,1,1,0 |  |  |  |  |  |  |
| Module10_25_77 | 0,0,1,0,1,0 |  |  |  |  |  |  |

**Additional Data File 8. Multiply-supported Subnetworks (MSSNs)**
