# Supplementary material for: A global analysis of genetic interactions in Caenorhabditis elegans
Source: J Biol. 2007 Sep 26;6(3):8. doi: 10.1186/jbiol58 (PMC2373897; doi:10.1186/jbiol58)
Supplement: Additional data file 10 — 33 focused subnetwork pairs are listed along with the corresponding enrichment of SGI links that bridge them. [file jbiol58-S10.doc]

| **Subnetwork Pair *** | **Observed Bridges** | **Expected Bridges** | **Enrichment** |
| --- | --- | --- | --- |
| E83, I51 | 3 | 0.53 | 5.67 |
| E83, P21 | 3 | 0.53 | 5.67 |
| P0, P13 | 3 | 0.57 | 5.30 |
| E54, P13 | 3 | 0.68 | 4.43 |
| I51, P7 | 5 | 1.15 | 4.35 |
| P21, P7 | 5 | 1.15 | 4.35 |
| I51, P13 | 5 | 1.19 | 4.21 |
| E172, I126 | 4 | 1.02 | 3.94 |
| E172, P6 | 4 | 1.02 | 3.94 |
| I51, P14 | 5 | 1.27 | 3.94 |
| P14, P21 | 5 | 1.27 | 3.94 |
| P13, P7 | 5 | 1.28 | 3.89 |
| I126, P7 | 3 | 0.79 | 3.81 |
| P6, P7 | 3 | 0.79 | 3.81 |
| P13, P21 | 6 | 1.63 | 3.67 |
| E172, I51 | 4 | 1.12 | 3.58 |
| E172, P21 | 4 | 1.12 | 3.58 |
| E160, P13 | 3 | 0.85 | 3.55 |
| P22, P7 | 3 | 0.85 | 3.53 |
| E102, I126 | 6 | 1.71 | 3.51 |
| E102, P6 | 6 | 1.71 | 3.51 |
| P14, P22 | 3 | 0.90 | 3.34 |
| P13, P8 | 7 | 2.11 | 3.32 |
| I34, P8 | 8 | 2.43 | 3.30 |
| I34, P13 | 4 | 1.26 | 3.17 |
| E68, I126 | 4 | 1.28 | 3.12 |
| E68, P6 | 4 | 1.28 | 3.12 |
| I34, P21 | 3 | 0.97 | 3.08 |
| I51, P8 | 5 | 1.69 | 2.96 |
| P21, P8 | 5 | 1.69 | 2.96 |
| E68, P13 | 4 | 1.50 | 2.67 |
| E68, P22 | 4 | 1.50 | 2.67 |
| E102, P22 | 5 | 1.97 | 2.53 |

**Additional Data File 10. Bridged Subnetwork Pairs**

***E = Co-expression, P = Co-phenotype, I = Interolog**
