# Supplementary material for: A global analysis of genetic interactions in Caenorhabditis elegans
Source: J Biol. 2007 Sep 26;6(3):8. doi: 10.1186/jbiol58 (PMC2373897; doi:10.1186/jbiol58)
Supplement: Additional data file 11 — The bridging propensity of various data types is represented. [file jbiol58-S11.doc]

| **Link Type** | **Bridging Links** | **Within Links** | **Observed^1^** | **Expected^2^** | **Bridging Propensity ^3,4^** |
| --- | --- | --- | --- | --- | --- |
| SGI | 612 | 78 | 0.89 | 0.83 | 7% * |
| Transposed Sga | 2,107 | 1,126 | 0.65 | 0.65 | 0% |
| Lehner 2006 | 150 | 44 | 0.77 | 0.82 | - 6% |
| Co-expression | 3,456 | 5,660 | 0.38 | 0.64 | - 41% **^, 5^ |
| Co-phenotype | 2,388 | 1,241 | 0.66 | 0.74 | - 11% **^, 5^ |
| Li 2004 | 492 | 99 | 0.83 | 0.89 | - 7% *^, 5^ |

^1^ Bridging ratio for linked gene pairs (Bridging Links / (Bridging Links + Within Links))

^2^ Bridging ratio for all tested gene pairs

^3^ (Observed / Expected) - 1

^4^ ‘*’ indicates P-value < e-4, ‘**’ indicates P-value < e-25

^5^ Significantly under-enriched

**Additional Data File 11. Comparison of bridging propensities**

All links for each data type were classified as within links (fall within any subnetwork), bridging links (link across two or more subnetworks without falling within any subnetwork), or neither.
