# Supplementary material for: A global analysis of genetic interactions in Caenorhabditis elegans
Source: J Biol. 2007 Sep 26;6(3):8. doi: 10.1186/jbiol58 (PMC2373897; doi:10.1186/jbiol58)
Supplement: Additional data file 13 — Precision levels of networks created using various cutoffs of the LOFA and PCC scores are plotted against network size. The arrow indicates the chosen co-phenotype network variant. [file jbiol58-S13.doc]

**Additional Data File 13: Comparison of co-phenotype network construction methods**

Network precision, as measured by GoProcess1000, as a function of network size. Arrow indicates size and precision of chosen co-phenotype network variant.
