# Supplementary material for: A global analysis of genetic interactions in Caenorhabditis elegans
Source: J Biol. 2007 Sep 26;6(3):8. doi: 10.1186/jbiol58 (PMC2373897; doi:10.1186/jbiol58)
Supplement: Additional data file 14 — Evidence supporting the validity of using a normal approximation in the Z-transformation to estimate bridging significance. [file jbiol58-S14.doc]

**Additional Data File 14. Evidence Supporting the Validity of using a Normal Approximation in the Z-transformation to estimate bridging significance.**

We investigated the validity of using a normal approximation in the Z-test for bridged subnetwork pairs. We expect the distribution for the number of bridging pairs to follow a Hypergeometric distribution if the links are drawn independently. Since the number of links is large, the distribution can be approximated with a Binomial distribution, which assumes the links are drawn with replacement instead of without-replacement. For example, if *p* is the probability that a random link will connect a specific gene pair, then the number of links between two subnetworks of size *h* and *k* could be approximated with a Binomial having parameters *hk* and *p*. Rather than use the binomial distribution directly, we chose to estimate significance using an empirically generated distribution using randomly rewired networks to avoid biases in our estimates due to the study design. The design tested 11 queries against different sets of RNAi targets. Therefore, not all pairs were tested as the Binomial approximation would assume. However, as shown below, the empirical distribution still is well approximated by a normal, supporting the use of Z-scores to assess subnetwork bridging significance.

To assess the validity of using a normal assumption, we plotted the distribution of the number of bridges between each pair of significantly bridged subnetworks in 1,000 permuted SGI networks along with the corresponding normal distribution that we estimated **(see Figure A below)**. As evidenced from these plots, most empirical distributions (red) are well approximated by their corresponding normal distribution, especially for the values on the right tail with which we are most concerned. The deviances fall well within the expected range of error (data not shown).

To further illustrate this point, we directly compared the significance estimates of bridging of the normal approximation to an empirically derived approximation using simulation **(see Table A below)**. As the empirical distribution is discrete, we determined a range for its significance. The upper bound for the empirical significance was calculated as the fraction of 1,000 permuted SGI networks with the observed number of bridges between the corresponding subnetwork pair. The lower bound was calculated as the fraction with more than the observed number of bridges. For each significantly bridged subnetwork pair, we determined the relationship of the approximated normal significance to these bounds. We classified each relationship as either “Inflated” (more significant than the lower bound), “Within Bounds” (between the upper and lower bound), or “Conservative” (less significant than the upper bound). Of the 33 significantly bridged subnetwork pairs, 22 (67%) were within bounds, 6 (18%) were slightly conservative, and 5 (15%) were slightly inflated. Based on this evidence, we conclude that the normal approximation is sufficiently accurate for this analysis.

**Figure A: The normal approximation for bridged subnetwork pairs is valid**

Shown in red is the distribution of the number of bridges between each of 33 significantly bridged subnetwork pair across 1,000 permuted SGI networks. Shown in blue is the corresponding approximated normal distribution. The x-axis displays the bridge count (integer values ranging from zero to the maximum number of bridges between the corresponding subnetwork pair in 1,000 permuted SGI networks). The y-axis gives the frequency of the corresponding bridge count.

| **Subnetwork Pair** | **Observed Bridges** | **Normal Significance** | **Lower Bound** | **Upper Bound** | **Relationship** |
| --- | --- | --- | --- | --- | --- |
| I34, F8 | 8 | 6.9E-06 | 0.000 | 0.000 | Within Bounds |
| I51, F7 | 5 | 1.3E-05 | 0.000 | 0.000 | Conservative |
| F21, F7 | 5 | 1.3E-05 | 0.000 | 0.000 | Conservative |
| I51, F13 | 5 | 2.0E-05 | 0.000 | 0.002 | Within Bounds |
| F13, F21 | 6 | 3.1E-05 | 0.000 | 0.001 | Within Bounds |
| F13, F7 | 5 | 3.5E-05 | 0.000 | 0.000 | Conservative |
| F13, F8 | 7 | 5.4E-05 | 0.000 | 0.000 | Conservative |
| E83, I51 | 3 | 7.7E-05 | 0.000 | 0.006 | Within Bounds |
| E83, F21 | 3 | 7.7E-05 | 0.000 | 0.006 | Within Bounds |
| E102, I126 | 6 | 1.1E-04 | 0.000 | 0.000 | Conservative |
| E102, F6 | 6 | 1.1E-04 | 0.000 | 0.000 | Conservative |
| F0, F13 | 3 | 1.1E-04 | 0.000 | 0.003 | Within Bounds |
| I51, F14 | 5 | 1.3E-04 | 0.000 | 0.001 | Within Bounds |
| F14, F21 | 5 | 1.3E-04 | 0.000 | 0.001 | Within Bounds |
| E172, I126 | 4 | 4.3E-04 | 0.001 | 0.005 | Inflated |
| E172, F6 | 4 | 4.3E-04 | 0.001 | 0.005 | Inflated |
| E54, F13 | 3 | 5.7E-04 | 0.000 | 0.007 | Within Bounds |
| I34, F13 | 4 | 1.3E-03 | 0.000 | 0.011 | Within Bounds |
| E172, I51 | 4 | 1.5E-03 | 0.001 | 0.019 | Within Bounds |
| E172, F21 | 4 | 1.5E-03 | 0.001 | 0.019 | Within Bounds |
| I126, F7 | 3 | 1.6E-03 | 0.000 | 0.022 | Within Bounds |
| F6, F7 | 3 | 1.6E-03 | 0.000 | 0.022 | Within Bounds |
| F22, F7 | 3 | 1.7E-03 | 0.000 | 0.012 | Within Bounds |
| I51, F8 | 5 | 1.8E-03 | 0.002 | 0.014 | Inflated |
| F21, F8 | 5 | 1.8E-03 | 0.002 | 0.014 | Inflated |
| E68, I126 | 4 | 3.5E-03 | 0.003 | 0.019 | Within Bounds |
| E68, F6 | 4 | 3.5E-03 | 0.003 | 0.019 | Within Bounds |
| E160, F13 | 3 | 5.2E-03 | 0.002 | 0.038 | Within Bounds |
| I34, F21 | 3 | 5.8E-03 | 0.000 | 0.031 | Within Bounds |
| E102, F22 | 5 | 6.1E-03 | 0.002 | 0.025 | Within Bounds |
| F14, F22 | 3 | 6.4E-03 | 0.007 | 0.026 | Inflated |
| E68, F13 | 4 | 8.8E-03 | 0.003 | 0.034 | Within Bounds |
| E68, F22 | 4 | 9.4E-03 | 0.004 | 0.029 | Within Bounds |

**Table A: The normal approximation for bridged subnetwork pairs is valid**

Comparison of approximated normal significance and empirical significance for each significantly bridged subnetwork pair. Upper bound for empirical significance is the fraction of 1,000 permuted SGI networks with the observed number of bridges between the corresponding pair. Lower bound is the fraction with more than the observed number of bridges. The final column gives the relationship of the approximated normal significance to these bounds: “Inflated”, more significant than the lower bound; “Within Bounds”, between upper and lower bound; “Conservative”, less significant than the upper bound. Of the 33 significantly bridged subnetwork pairs, 22 (67%) are within the bounds, 6 (18%) are slightly conservative, and 5 (15%) are slightly inflated.
